# Heritability of cognitive and emotion processing during functional MRI in a twin sample

**DOI:** 10.1002/hbm.26557

**Published:** 2024-01-12

**Authors:** Haeme R. P. Park, Miranda R. Chilver, Yann Quidé, Arthur Montalto, Peter R. Schofield, Leanne M. Williams, Justine M. Gatt

**Affiliations:** ^1^ Neuroscience Research Australia Sydney New South Wales Australia; ^2^ School of Psychology University of New South Wales Sydney New South Wales Australia; ^3^ School of Biomedical Sciences University of New South Wales Sydney New South Wales Australia; ^4^ Psychiatry and Behavioral Sciences, Stanford School of Medicine Stanford University California USA

**Keywords:** attention, emotion, functional MRI, heritability, twin, working memory

## Abstract

Despite compelling evidence that brain structure is heritable, the evidence for the heritability of task‐evoked brain function is less robust. Findings from previous studies are inconsistent possibly reflecting small samples and methodological variations. In a large national twin sample, we systematically evaluated heritability of task‐evoked brain activity derived from functional magnetic resonance imaging. We used established standardised tasks to engage brain regions involved in cognitive and emotional functions. Heritability was evaluated across a conscious and nonconscious Facial Expressions of Emotion Task (FEET), selective attention Oddball Task, N‐back task of working memory maintenance, and a Go‐NoGo cognitive control task in a sample of Australian adult twins (*N* ranged from 136 to 226 participants depending on the task and pairs). Two methods for quantifying associations of heritability and brain activity were utilised; a multivariate independent component analysis (ICA) approach and a univariate brain region‐of‐interest (ROI) approach. Using ICA, we observed that a significant proportion of task‐evoked brain activity was heritable, with estimates ranging from 23% to 26% for activity elicited by nonconscious facial emotion stimuli, 27% to 34% for N‐back working memory maintenance and sustained attention, and 32% to 33% for selective attention in the Oddball task. Using the ROI approach, we found that activity of regions specifically implicated in emotion processing and selective attention showed significant heritability for three ROIs, including estimates of 33%–34% for the left and right amygdala in the nonconscious processing of sad faces and 29% in the medial superior prefrontal cortex for the Oddball task. Although both approaches show similar levels of heritability for the Nonconscious Faces and Oddball tasks, ICA results displayed a more extensive network of heritable brain function, including additional regions beyond the ROI analysis. Furthermore, multivariate twin modelling of both ICA networks and ROI activation suggested a mix of common genetic and unique environmental factors that contribute to the associations between networks/regions. Together, the results indicate a complex relationship between genetic factors and environmental interactions that ultimately give rise to neural activation underlying cognition and emotion.

## Graphical abstract

We used fMRI to determine the heritability of task‐evoked brain activity related to cognition and emotion using independent component analysis (ICA) and a region‐of‐interest approach. Although, both analyses resulted in similar levels of heritability, ICA displayed a more extensive network of heritable brain function. The influence of genetics and environment are differentially distributed across different functional domains, indicating a complex relationship between the two on neural phenotypes.

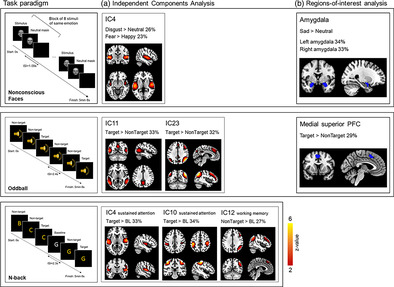

## 1 INTRODUCTION

Twin studies have been invaluable in examining the heritability of genetic contributions and environmental factors to a wide range of physical and psychological processes. Heritability measures the proportion of phenotypic variability that may be attributable to genetic factors, where higher estimates indicate a larger influence of genetic variability on a given trait within the population (Verweij et al., 2012). In classical twin studies, it is possible to decompose the variance of a given phenotypic trait into additive genetic factors (*A*; heritability), dominant genetic (*D*) or shared environment (*C*) effects, and non‐shared/unique environment (*E*), by modelling the trait correlations between monozygotic (MZ) and dizygotic (DZ) twins (Rijsdijk & Sham, 2002). Numerous behavioural studies have reported heritability for cognitive and executive function processes (Croston et al., 2015; Fan et al., 2001; Finkel et al., 1995; Zhu et al., 2010) and emotion processing (Routledge et al., 2017); however, twin studies utilising task‐based neuroimaging methods that probe such cognitive/emotion processes are more sparse as compared to resting state, task‐free, or brain structural methods, despite their potential contribution to our understanding of the influence of genetics on brain–behaviour relationships. To this end, the aim of the current study was to investigate the extent to which brain function elicited by executive function and emotion processing tasks may be heritable in the same sample of twins using functional magnetic resonance imaging (fMRI). We sought to determine the genetic and environmental contributions on task‐related functional networks that may underlie various behavioural phenotypes.

Twin studies have provided strong evidence for the heritability of brain morphology (e.g., grey matter volume, white matter integrity; Blokland et al., 2012; Brouwer et al., 2017; Den Braber et al., 2013), suggesting the variance in the brain structure of different regions can be attributed to genetics. On the other hand, heritability studies of brain function (e.g., as assessed by fMRI) are less common and with mixed findings. For example, within the emotion processing domain, a PubMed search with keywords ((emoti*[Title/Abstract]) AND (heritab*[Title/Abstract])) AND (fmri[Title/Abstract] OR mri[Title/Abstract]) resulted in only 26 publications of which the majority examine resting state or intrinsic (task‐free) networks or use structural MRI methodology, or focus on associations between candidate genes (e.g., SNPs) and brain activity, rather than genetic modelling. Methodological differences between studies may have further contributed to this discrepancy, including differences in tasks/processes probed (e.g., emotional vs. attentional processes) and MRI methods employed (e.g., whole‐brain vs. region‐of‐interest approaches).

Out of the limited number of studies, working memory processes have received much attention within the executive functioning domain, with multiple studies reporting significant heritability (ranging from 34% to ~80%) for functional activity in the frontal lobe, supplementary motor area, pre‐ and postcentral gyri, and regions in the parietal lobe (Blokland et al., 2008, 2011; Etzel et al., 2020; Koten Jr et al., 2009). Heritability of brain function has also been reported for other cognitive processes, such as conflict processing (e.g., anterior cingulate cortex; Matthews et al., 2007), reward processing (e.g., nucleus accumbens; Li et al., 2019), and visual processing (e.g., visual cortex; Park et al., 2012; Polk et al., 2007), as well as for canonical neural networks (e.g., the default mode, salience, executive control) derived from resting state data (Adhikari et al., 2018; Korgaonkar et al., 2014; Yang et al., 2016). In contrast, within the emotion processing domain, a previous fMRI study has indicated no evidence of genetic influences for brain regions involved in processing sad emotions (medial and ventrolateral prefrontal cortex; Côté et al., 2007), despite behavioural and electroencephalography (EEG) studies showing heritability for facial emotion processing that ranges from 27% to 37% for behavioural reaction times (Routledge et al., 2018), and 46% to 64% for event‐related potentials in response to emotional faces (Anokhin et al., 2010).

Another reason for the variability in results may be due to the statistical methodology used. Most previous studies have used univariate statistics for *a priori* determined regions‐of‐interest (ROI; e.g., Blokland et al., 2008; Li et al., 2018). Although this hypothesis driven approach has strengths compared to the mass univariate whole‐brain analyses (including stronger power due to not having to correct for 100,000s voxels in the brain), it can limit the identification of brain function heritability to these specific regions. As genetic and environmental influences on brain function are likely to affect task‐related networks, two studies have recently utilised multivariate pattern similarity analysis to compare patterns of brain function in twins related to cognitive control processes, specifically within the frontoparietal network (Etzel et al., 2020; Tang et al., 2021) and noted the increased power to detect heritability of task‐based fMRI activity using this technique. Therefore, using a multivariate method may be a more powerful approach to investigate the genetic influence on brain function.

The current study uses fMRI data collected from the large TWIN‐E cohort study consisting of 1669 healthy Australian twin adults, of which MRI data was obtained from a subset of 263 participants (for more detail regarding the TWIN‐E project, please see Gatt et al., 2012). This study examined the contribution of genetic and environmental factors on task‐related functional MRI networks in these twin participants. Five experimental tasks spanning both emotion and cognitive domains were analysed (conscious and nonconscious Facial Expressions of Emotion, Oddball, N‐back, and Go‐NoGo tasks), providing a unique opportunity to compare heritability estimates across different paradigms in the same cohort of twins. We hypothesised that functional networks related to working memory and attention would show moderate heritability, especially in frontoparietal regions that are typically engaged in cognitive control (Etzel et al., 2020; Niendam et al., 2012) and emotion processing (Anokhin et al., 2010; Routledge et al., 2018).

Additionally, both data‐driven (using independent component analysis; ICA) and hypothesis‐driven (ROI) methods were used to determine the brain regions associated with genetic heritability across these five tasks, while a behaviour genetics approach was adopted for twin modelling. As the ICA method consists of decomposing fMRI signals into independent sources that can be used to identify task‐correlated networks, we hypothesised that this method would obtain more widespread areas of activation, when compared to the ROI method that is constrained to discrete anatomical regions that are chosen a priori. The outcomes from this study provide an overview of genetic vs. environmental contributions to common cognitive and emotion processes, and shed light on the utility of two different analytical methods in future heritability‐imaging studies.

## 2 METHODS AND MATERIALS

### 2.1 Participants

The sample consisted of 263 participants (175 monozygotic and 88 dizygotic healthy same‐sex twins) who completed the MRI component of the larger TWIN‐E project (see Gatt et al., 2012). In addition, participants were screened for current and previous mental and physical health history, as well as suitability for MRI. The study received approval from the Human Research Ethics Committee of the University of Sydney (03‐2009/11430), and written informed consent was sought from all participants prior to data collection.

### 2.2 fMRI paradigms

The MRI component consisted of five functional MRI tasks, as well as a structural scan, a diffusion‐weighted scan, and a proton density scan. For the purposes of this study, only the functional scans are examined here. The tasks employed spanned emotion and cognitive domains, including: (1) a Nonconscious Facial Expressions of Emotion task, which examined subliminal processing of emotional faces; (2) a Conscious Facial Expressions of Emotion task, which examined conscious processing of emotional faces; (3) a N‐back task, which probed both working memory and sustained attention; (4) a Go‐NoGo task, which examined response inhibition; and (5) an Oddball task, which examined responses related to selective attention (for a detailed description of the tasks, see Appendix S1.1 and Gatt et al., 2012).

### 2.3 Image acquisition and preprocessing

Details on imaging acquisition parameters and preprocessing of fMRI data are provided in Appendix S1.2.

### 2.4 Independent component analyses

First, all preprocessed functional images were checked for quality control, and matched for twinness (i.e., if one twin failed quality control, both twins were dropped from further analyses including heritability analysis). Missing functional images and signal drop out were the main reasons for excluding participants. Due to equipment failure (a drop out in communication between the MRI response boxes and the experimental computer) during some of the MRI sessions, no behavioural data were recorded for approximately a third of the participants. Thus, for the N‐back and Go‐NoGo task, only participants with usable behavioural data (in order to confirm accurate working memory/sustained attention processes and successful inhibition) were included in the analyses (Table 1). We then performed spatial independent component analysis (ICA) on the functional data using the Group ICA of fMRI Toolbox (GIFT; http://icatb.sourceforge.net) implemented in Matlab R2018b (see Appendix S1.3 for more detail).

**TABLE 1:** fMRI parameters for the five functional tasks.

Task	Total sample (*n*)	Task regressors	ICA		ROI analyses	
			Number of ICs	Contrasts‐of‐interest	ROIs tested	Beta estimate differences
Nonconscious Facial Expressions of Emotion	226	Angry, Happy, Fear, Disgust, Sad, Neutral	27	Each emotion > Neutral; each negative emotion > Happy	*Angry/Fear*: dACC, bilateral amygdala	Angry > Neutral; Fear > Neutral
					*Sad*: pgACC, bilateral insula, bilateral amygdala	Sad > Neutral
					*Happy*: vmPFC, bilateral striatum	Happy > Neutral
Conscious Facial Expressions of Emotion	220	Angry, Happy, Fear, Disgust, Sad, Neutral	27	Each emotion > Neutral; each negative emotion > Happy	*Angry/Fear*: dACC, bilateral amygdala	Angry > Neutral; Fear > Neutral
					*Sad*: pgACC, bilateral insula, bilateral amygdala	Sad > Neutral
					*Happy*: vmPFC, bilateral striatum	Happy > Neutral
N‐back	168	Working Memory, Sustained Attention, Baseline	26	Target ≠ NonTarget ≠ Baseline	*Working memory*: dACC, bilateral dlPFC	NonTarget > Baseline (testing for working memory)
					*Attention*: msPFC, bilateral LPFC, bilateral aIPL, bilateral precuneus	Target > Baseline (testing for sustained attention)
Oddball	220	Target, NonTarget	27	Target > NonTarget	msPFC, bilateral LPFC, bilateral aIPL, bilateral precuneus	Target > NonTarget
Go‐NoGo	136	Correct NoGo, Correct Go, Failed NoGo, Errors	22	Correct NoGo ≠ Correct Go ≠ Failed NoGo	dACC, bilateral dlPFC	Correct NoGo > Correct Go

*Note*: Total number of the sample reflects the final sample of participants for each task included in all analyses (ICA, ROI, and twin modelling).

Abbreviations: aIPL, anterior inferior parietal lobule; dACC, dorsal anterior cingulate cortex; dlPFC, dorsolateral prefrontal cortex; IC, independent component; ICA, independent components analysis; LPFC, lateral prefrontal cortex; msPFC, medial superior prefrontal cortex; pgACC, pregenual anterior cingulate cortex; ROI, region‐of‐interest; vmPFC, ventromedial prefrontal cortex; > indicate directional *t*‐tests, ≠ indicate one‐way ANOVAs.

To identify the independent components (ICs) that were significantly associated with the task, design matrices of task regressors were constructed using SPM12 for each of the five tasks separately. For each matrix, there were six regressors for both the Nonconscious and Conscious Facial Expressions of Emotion tasks (FEET; Angry, Happy, Fear, Disgust, Sad, and Neutral), four for Go‐NoGo (Correct NoGo, Correct Go, Failed NoGo, Other errors), three for the N‐back (Target, NonTarget, Baseline), and two for the Oddball (Target, NonTarget). These matrices were then included in a multiple linear regression within GIFT to provide a reference time course for each task. Beta weights for each regressor per participant were then created, which indicated positive and negative correlations of the observed time course within each component to the reference. Due to its data driven nature, significance of each component derived from ICA was tested by running one‐way ANOVAs on these beta weights for each component for the N‐back and Go‐NoGo tasks, and post‐hoc *t*‐tests were conducted if the ANOVA was significant. For the Oddball and the Nonconscious and Conscious conditions of the FEET, we chose to run paired *t*‐tests as these tasks had specific *a priori* contrasts regarding brain activation (e.g., we wanted to examine regions associated with specific emotion processing, and therefore chose to contrast each emotion against neutral; see Table 1). For each task, we applied Bonferroni correction to account for multiple testing using the number of ICs tested (e.g., correcting for 26 tests for N‐back and 22 for Go‐NoGo).

### 2.5 ROI analyses

For the nonconscious and conscious conditions of the Facial Expressions of Emotion Task, the blood‐oxygen level‐dependent (BOLD) responses were modelled using a boxcar stimulus function, while for the N‐back, Oddball, and Go‐NoGo tasks, BOLD responses were modelled by delta functions at stimulus onset, due to the event‐related nature of these three paradigms (SPM12 manual, www.fil.ion.ucl.ac.uk/spm/doc/manual.pdf). A canonical haemodynamic response function was applied to the onsets, resulting in time courses that were applied to a general linear model for each task. A high‐pass filter with a cut‐off frequency of 128 s was used to remove low‐frequency noise, and an AR(1) model was used to estimate serial autocorrelations. Following first‐level (individual) modelling of the tasks (see Appendix S1.5), average task‐related signal (beta weight parameters) within ROIs from circuits related to cognitive control, attention, and affect processes derived from Neurosynth.org (https://www.neurosynth.org/; Goldstein‐Piekarski et al., 2022) were extracted with the MarsBar toolbox (http://marsbar.sourceforge.net/) for focal analyses (see Table 1 and Appendix S1.4 for more detail on ROI selection). Due to the specificity of the networks, we restricted our contrasts to Angry > Neutral, Fear > Neutral, Sad > Neutral, and Happy > Neutral for the Faces tasks. For the cognitive tasks, we also went directly to our hypothesised *t*‐contrasts due to the *a priori* nature of the ROIs which were chosen for their involvement in cognitive processes. As we did not model group‐level contrasts, the beta estimate differences for each participant were taken directly to heritability analyses. Similar to the ICA approach, for each task, we applied Bonferroni correction to account for the number of ROIs tested, adjusting for 3 ROIs for the Go‐NoGo task and the working memory contrast of the N‐back task (*p‐corr* = 0.017) and 7 for the Oddball task and the sustained attention contrast of the N‐back task (*p‐corr* = 0.007).

### 2.6 Univariate twin analyses

The beta weight parameters from ICs that were significantly associated with each task were used in univariate twin analyses to determine the heritability of the task‐related functional networks. This was also done for the ROI analyses, where the beta estimate differences were used to examine heritability of specific regions identified in neural circuits related to emotion and cognition (Goldstein‐Piekarski et al., 2022; Williams, 2016). We adopted a classical twin design for our univariate modelling analysis (Boomsma et al., 2002; Røysamb & Tambs, 2016), which is based on the comparison of the twin‐cotwin similarity across monozygotic (MZ) versus dizygotic (DZ) pairs. Initial assumption testing showed that constraining means and variances across twin 1 and twin 2, as well as across MZ and DZ twin pairs did not result in a worse model fit compared to the fully saturated model, therefore meeting the basic data assumptions for classical twin modelling analyses. If the intra‐class correlation between MZ twins was double (or more) than the intra‐class correlation between DZ twins, an ADE model was chosen as the starting model, otherwise an ACE model was tested (Verweij et al., 2012; see Table S2.2 for more detail). Structural equation models were run using the *OpenMx* package (version 2.19.5; https://openmx.ssri.psu.edu/docs/OpenMx/latest; Neale et al., 2016) in *R*, where additive genetic (*A*), non‐additive genetic (*D*), shared environment (*C*), and non‐shared environment (*E*) variance components were examined using the beta parameters derived from the ICA and ROI analyses (see Table 1). Age and sex were included as covariates. To test whether there was a significant heritable component in each network, the full univariate ACE/ADE model was contrasted with its submodels (AE, CE/DE, and E) by removing either one or two of the variance components. The model fit was determined by looking at the *χ* ^2^ goodness‐of‐fit, where a significant difference between the models indicated a decrease in model fit.

### 2.7 Multivariate twin analyses

Lastly, we ran two multivariate twin models to examine whether there was any evidence of shared and non‐shared genetic and environmental variance across the components/regions that showed significant heritability in the univariate analyses. In more detail, two separate correlated factors models were run to calculate the covariance between either the significant heritable components (from ICA) or the significant heritable regions (from ROI analysis) using *OpenMx* in R. For this analysis, each phenotype (or variable; i.e., extracted parameters from either ICA or ROI analysis) is decomposed into its genetic and unique environmental components, whereby the correlations across these phenotypes are then estimated (Posthuma, 2009). A common genetic influence is indicated when there is a significant genetic correlation (*r*_A_) between two variables, while a significant environmental correlation (*r*_E_) would be driven by unique environmental factors. Age and sex were again included as covariates. Genetic and environmental correlations between the components/regions were tested by constraining each correlation to zero in a new model, which was then compared to the previous model and checked for model fit. Similar to univariate twin modelling, this was tested by using the *χ* ^2^ goodness‐of‐fit, as well as checking the 95% confidence intervals.

## 3 RESULTS

### 3.1 Sample characteristics

From the initial 263 participants, 13 participants were excluded due to being a single twin (i.e., their twin did not participate in the MRI session). Out of 250 participants who completed the MRI session, those who failed quality control (i.e., signal drop‐out, missing fMRI data) and their matched twins were excluded from further analysis. For both N‐back and Go‐NoGo tasks, participants with missing behavioural data (mainly due to equipment recording failure) or whose number of errors was greater than 2 SD from the mean were excluded from analyses, resulting in a reduced sample. Sample size and other demographic characteristics are summarised in Table 2 and Table S2.3.

**TABLE 2:** Demographic characteristics of the samples included in the fMRI and twin modelling analyses.

Characteristic	Nonconscious FEET (*n* = 226)	Conscious FEET (*n* = 220)	N‐back (*n* = 168)	Oddball (*n* = 218)	Go‐NoGo (*n* = 136)
Zygosity (MZ/DZ)	154/72	150/70	102/66	150/68	86/50
Age (years ± SD)	38.9 (±12.5)	38.8 (±12.6)	39.6 (±13.0)	39.0 (±12.5)	38.6 (±12.6)
Sex (M/F)	86/140	82/138	66/102	82/136	50/86

Abbreviations: DZ, dizygotic twin; FEET, Facial Expressions of Emotion Task; M/F, male/female; MZ, monozygotic twin; SD, standard deviation.

### 3.2 ICA results

A complete list of ICs found for each task and the brain regions within each component are reported in Tables S2.1.1 ‐ S2.1.5. From these, only the components specifically associated with each task design and contrast (see Appendix S2.1) were used in heritability analyses.

### 3.3 Univariate twin modelling results

**FIGURE 1:**
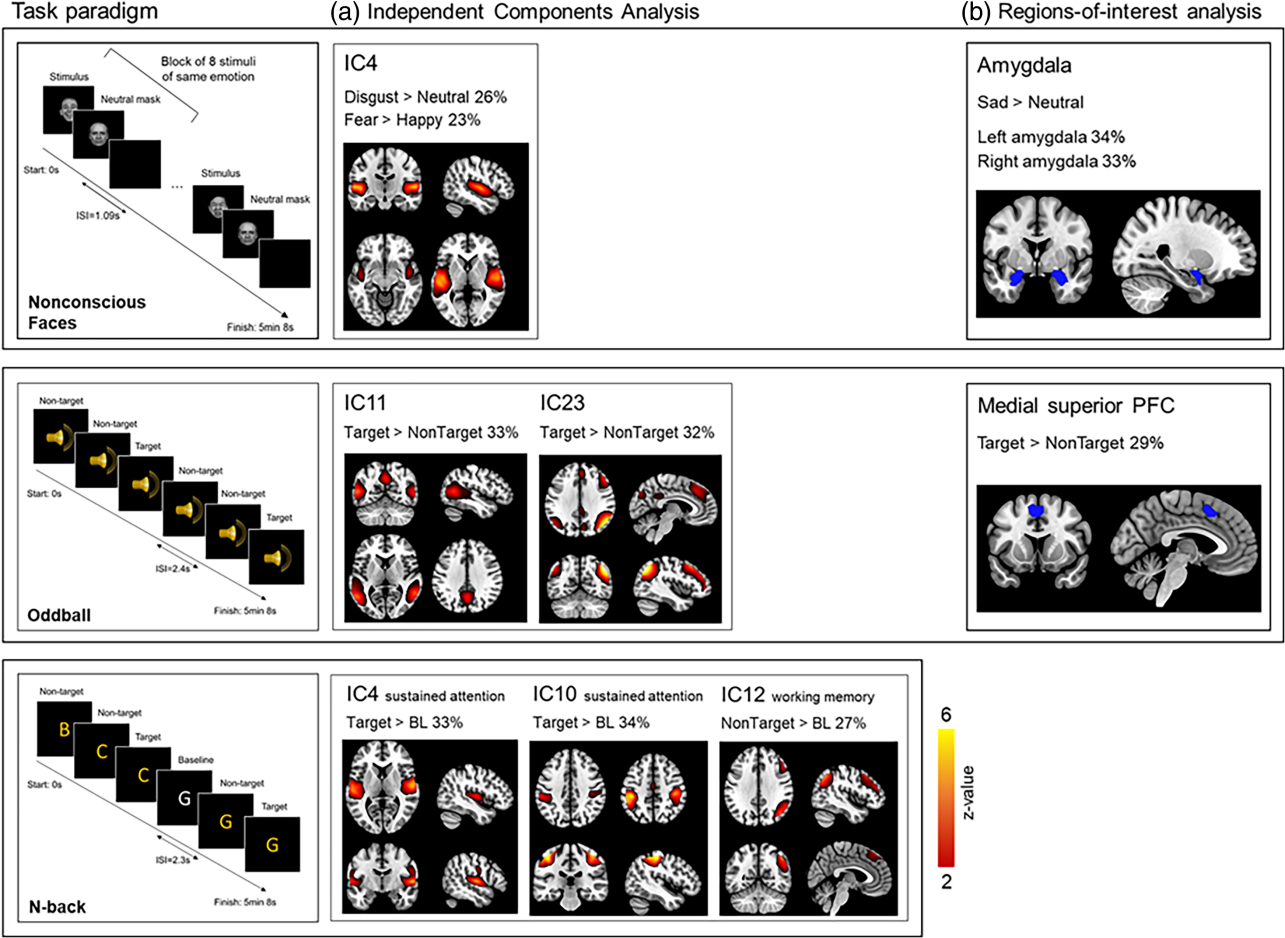
(a) Spatial maps of the six independent components (IC) associated with Nonconscious Facial Expressions of Emotion Task (FEET), Oddball, and N‐back tasks that showed a significant genetic basis during twin modelling analyses. For the Nonconscious FEET, the regions in IC4 included the superior temporal gyrus and the insula. For Oddball, IC11 included middle and superior temporal gyri while IC23 included middle frontal and superior frontal gyri, middle temporal gyrus, supramarginal gyrus, angular gyrus, and the inferior parietal lobule. For N‐back, IC4 included superior temporal gyrus, precentral gyrus, and the insula, IC10 included pre‐ and postcentral gyri and the inferior parietal lobule, and IC12 included middle and superior frontal gyri, supramarginal gyrus, and the inferior parietal lobule. The components were thresholded at |*z*| > 3.5. For the component that correlated with multiple contrasts, each contrast and its associated heritability is listed (i.e., IC4 for Nonconscious Faces). (b) Regions‐of‐interest that showed significant heritability for the Nonconscious Faces task (bilateral amygdala) and the Oddball (medial superior prefrontal cortex; PFC). Images are in neurological convention (left side of the image corresponds to the left hemisphere); BL, baseline; ISI, interstimulus interval.

For each task, we report heritability using ICA and then ROI‐based approaches. Only the independent components and the regions‐of‐interest that show significant heritability are reported below (see Tables 3 and 4; Figure 1).

**TABLE 3:** Univariate heritability estimates for the ICA task‐related networks that show significant heritability.

Task	IC	Contrast	Model	Model fit					Parameter estimates		
				Comparison	−2LL	AIC	df	*p*	*A* [CI]	*C* [CI]	*E* [CI]
Noncon FEET	4	Disgust > Neutral	ACE	vs. saturated	282.71	−157.29	220	‐	0	0.242 [0, 0.41]	0.758 [0.59, 0.94]
			**AE**	**vs. ACE**	**283.75**	**−158.25**	**221**	**.309**	**0.255 [0.05, 0.44]**	**0**	**0.745 [0.56, 0.95]**
			E	vs. AE	289.53	−154.47	222	.016			1
	4	Fear > Happy	ACE	vs. saturated	300.85	−139.15	220	‐	0.069 [0, 0.41]	0.153 [0, 0.38]	0.778 [0.59, 0.96]
			**AE**	**vs. ACE**	**300.98**	**−141.02**	**221**	**.718**	**0.231 [0.03, 0.41]**	**0**	**0.769 [0.59, 0.97]**
			E	vs. AE	306.19	−137.81	222	.022			1
N‐back	4	Target > Baseline	ACE	vs. saturated	635.46	647.46	162	‐	0	0.350 [0.15, 0.52]	0.650 [0.48, 0.85]
			**AE**	**vs. ACE**	**638.83**	**648.83**	**163**	**.067**	**0.326 [0.10, 0.51]**	**0**	**0.673 [0.49, 0.90]**
			E	vs. AE	646.47	654.47	164	.006			1
	10	Target > Baseline	ACE	vs. saturated	660.10	672.10	162	‐	0.335 [0, 0.54]	0.004 [0, 0.45]	0.661 [0.46, 0.91]
			**AE**	**vs. ACE**	**660.10**	**670.10**	**163**	**.992**	**0.339 [0.10, 0.54]**	**0**	**0.661 [0.46, 0.90]**
			E	vs. AE	667.40	675.40	164	.007			1
	12	NonTarget > Baseline	ACE	vs. saturated	663.16	675.16	160	‐	0.269 [0, 0.49]	0	0.731 [0.51, 0.98]
			**AE**	**vs. ACE**	**663.16**	**673.16**	**161**	**1.00**	**0.269 [0.02, 0.49]**	**0**	**0.731 [0.51, 0.98]**
			E	vs. AE	667.47	675.47	162	.038			1
Oddball	11	Target > NonTarget	ACE	vs. saturated	870.63	450.63	210	‐	0.323 [−0.37, 0.50]	0	0.675 [0.50, 0.89]
			**AE**	**vs. ACE**	**870.63**	**448.63**	**211**	**1.00**	**0.326 [0.12, 0.50]**	**0**	**0.674 [0.50, 0.88]**
			E	vs. AE	879.75	455.75	212	.003			1
	23	Target > NonTarget	ACE	vs. saturated	910.07	490.07	210	‐	0	0.367 [−0.28, 0.55]	0.633 [0.45, 0.85]
			**AE**	**vs. ACE**	**912.02**	**490.02**	**211**	**.163**	**0.323 [0.11, 0.51]**	**0**	**0.678 [0.49, 0.89]**
			E	vs. AE	920.29	496.29	212	.004			1

*Note*: The variance was decomposed into additive (*A*; heritability), common (*C*), and unique environmental (*E*) factors as the intra‐class correlations for MZ were less than twice the DZ intra‐class correlations (suggesting a ‘*C*’ over ‘*D*’ component to test; see Table S2.2). Models highlighted in bold were selected as the best‐fitting models based on parsimony and significant deterioration of model fit after dropping a parameter. The regions comprised in each IC (independent component) are illustrated in Figure 1.

Abbreviations: −2LL, minus twice the log likelihood; AIC, Akaike's information criterion; CI, 95% confidence intervals; df, degrees of freedom; IC, independent component; Noncon FEET, Nonconscious Facial Expressions of Emotion Task.

**TABLE 4:** Estimated heritability contributions to ROI activity evoked by cognitive and emotion tasks.

Task	ROI	Contrast	Model	Model fit					Parameter estimates		
				Comparison	−2LL	AIC	df	*p*	*A* [CI]	*D* [CI]	*E* [CI]
Noncon FEET	L amygdala	Sad > Neutral	ADE	vs. saturated	−169.33	−157.33	220	‐	0	0.213 [0, 0.53]	0.652 [0.47, 0.86]
			**AE**	**vs. ADE**	**−169.21**	**−159.22**	**221**	**.735**	**0.337 [0.13, 0.51]**	**0**	**0.663 [0.49, 0.87]**
			E	vs. AE	−159.60	−151.60	222	.002			1
	R amygdala	Sad > Neutral	ADE	vs. saturated	−182.81	−170.81	220	‐	0	0.359 [0, 0.53]	0.641 [0.47, 0.85]
			**AE**	**vs. ADE**	**−181.51**	**−171.51**	**221**	**.255**	**0.328 [0.12, 0.51]**	**0**	**0.672 [0.49, 0.88]**
			E	vs. AE	−172.46	−164.46	222	.003			1
Oddball	Medial superior PFC	Target > NonTarget	ADE	vs. saturated	691.73	703.73	212	‐	0	0.310 [0, 0.49]	0.690 [0.51, 0.90]
			**AE**	**vs. ADE**	**692.22**	**702.22**	**213**	**.481**	**0.292 [0.08, 0.48]**	**0**	**0.708 [0.52, 0.92]**
			E	vs. AE	699.51	707.51	214	.007			1

*Note*: The variance was decomposed into additive (*A*; heritability), non‐additive (*D*), and unique environmental (*E*) factors as the intra‐class correlations for MZ were double or more compared to the DZ intra‐class correlations (suggesting a ‘*D*’ over ‘*C*’ component to test; see Table S2.2). Models highlighted in bold were selected as the best‐fitting models based on parsimony and significant deterioration of model fit after dropping a parameter.

Abbreviations: −2LL, minus twice the log likelihood; AIC, Akaike's information criterion; CI, 95% confidence intervals; df, degrees of freedom; L, left; Noncon FEET, Nonconscious Facial Expressions of Emotion Task; PFC, prefrontal cortex; R, right; ROI, region‐of‐interest.

#### 3.3.1 ICA approach

##### Conscious facial emotion stimuli

There was no significant contribution of heritability for ICA‐derived functional network components derived for conscious facial emotion stimuli.

##### Nonconscious facial emotion stimuli

We observed a significant contribution of heritability to the ICA‐derived network 4 (consisting of the superior temporal gyrus and insula) elicited by nonconscious disgust relevant to neutral stimuli (26%, *p* = .016; Table 3). The same network was also associated with the nonconscious fear relative to happy stimuli, again showing significant heritability (23%, *p* = .022; Table 3).

Heritability did not make a significant contribution to any other ICA‐derived brain function networks for the nonconscious facial emotion stimuli.

##### N‐back

For the ICA‐derived networks related to working memory (NonTarget > Baseline), we found no significant heritability (*p* > .05) of the functional network represented in IC4 (superior temporal and precentral gyri, insula) and IC14 (fronto‐parieto‐temporal network). For IC12 (fronto‐parietal regions, including the inferior parietal lobule), a significant heritability estimate was found (27%, *p* = .038; Table 3) with the AE model being the best fitting model.

For the networks related to the sustained attention (Target > Baseline) contrast, components IC4 (superior temporal and precentral gyri, insula) and IC10 (pre‐ and postcentral gyri, inferior parietal lobule) both showed significant heritability (IC4: 33%, *p* = .006; IC10: 34%, *p* = .007; Table 3), again with the AE being the best fitting model for both components. No significant heritability was found for IC14 (*p* > .05).

##### Oddball

For the eight components related to novelty processing (Oddball Target > Baseline), we observed significant heritability for IC11 (superior and middle temporal gyri, 33%; *p* = .003) and IC23 (fronto‐parieto‐temporal network, 32%; *p* = .004), where an AE model was the best fitting model. No other significant heritability was observed for the remaining components (*p* > .05).

##### Go‐NoGo

There was no significant contribution of heritability for ICA‐derived brain function network components underlying response inhibition elicited by NoGo relevant to Go stimuli (*p* > .05).

#### 3.3.2 ROI approach

##### Conscious facial emotion stimuli

There was a trend towards a contribution from heritability to activity of the ventral striatum elicited by happy relative to neutral faces (20%, *p* = .048 uncorrected).

##### Nonconscious facial emotion stimuli

Our AE model revealed that there was a significant contribution from heritability to activity of the bilateral amygdala elicited by nonconscious sad relative to neutral face stimuli (left: 34%, *p* = .002; right: 33%, *p* = .003; Table 4) but not for other emotion stimuli (*p* > .05).

##### N‐back

There was no significant contribution of heritability in regions of activity for the N‐back task.

##### Oddball

In the AE model, there was a significant contribution of heritability to Oddball target‐elicited medial superior prefrontal cortical activity (29%, *p* = .007; Table 4).

##### Go‐NoGo

There was no significant contribution of heritability in regions of activity for the Go‐NoGo task.

### 3.4 Multivariate twin modelling results

#### 3.4.1 ICA model

**FIGURE 2:**
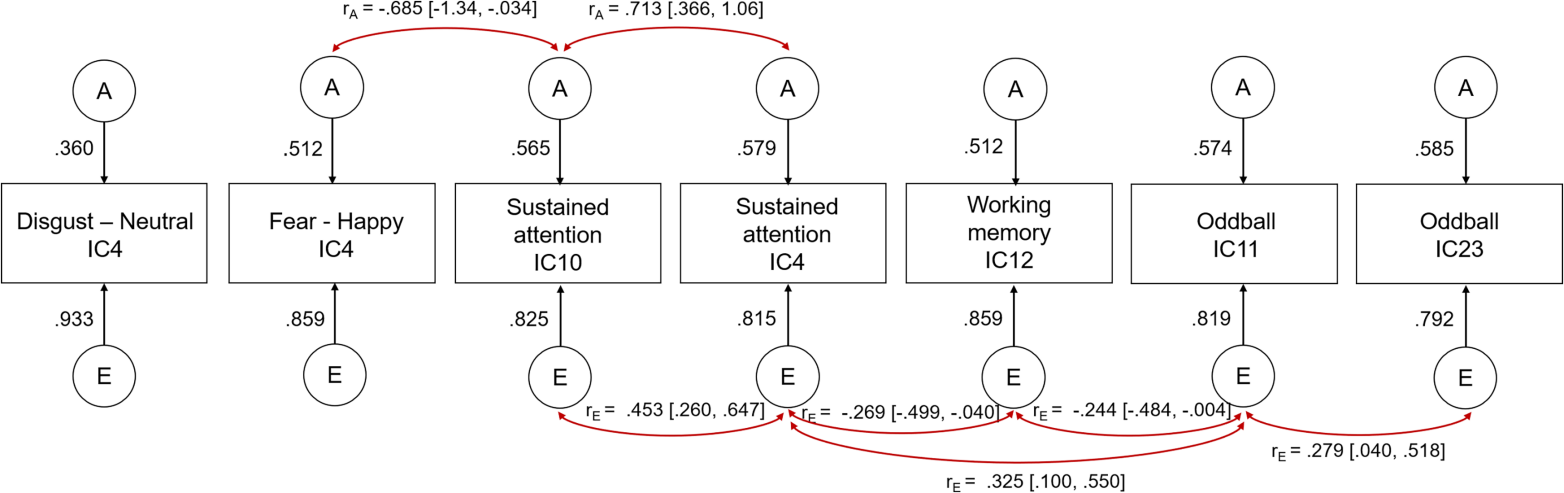
An AE correlated factors model for the seven independent components/networks derived from the ICA analyses that showed significant heritability. Only significant correlations are shown.

From our multivariate model with seven independent components that showed heritability, we identified significant genetic correlations between one of the components from the nonconscious Facial Expressions of Emotion task (Fear > Happy) IC4 and the sustained attention‐related component IC10 (*r*_A_ = −0.685, *p* = .026), and also between the two different sustained attention components IC10 and IC4 (*r*_A_ = 0.713, *p* = .014; Figure 2). We further observed significant unique environmental correlations between the two sustained attention‐related networks IC10 and IC4 (*r*_E_ = 0.453, *p* < .001), between sustained attention IC4 and working memory IC12 (*r*_E_ = −0.269, *p* = .029), between sustained attention IC4 and selective attention (Oddball) IC11 (*r*_E_ = 0.325, *p* = .009), between working memory IC12 and selective attention (Oddball) IC11 (*r*_E_ = −0.244, with a marginal *p* = .058), and between the two selective attention (Oddball) networks IC11 and IC23 (*r*_E_ = 0.279, *p* = .030).

#### 3.4.2 ROI model

**FIGURE 3:**
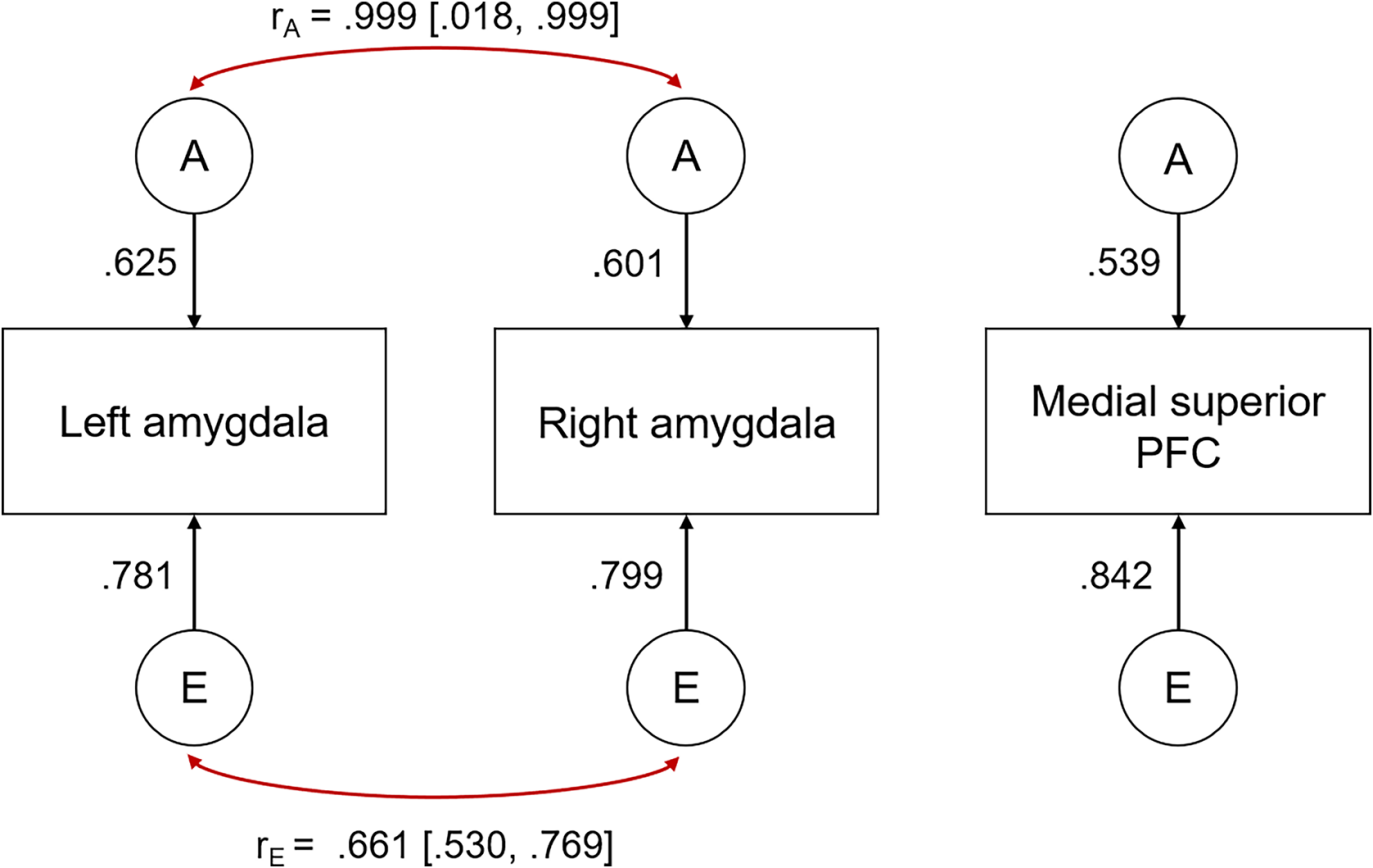
An AE correlated factors model for the three regions of interest from the ROI analyses that showed significant heritability. Only significant correlations are shown.

Finally, we ran a second correlated factors model with the three regions that showed heritability, which revealed a very high genetic correlation between the bilateral amygdala (*r*_A_ = 0.999, *p* < .001), as well as a significant unique environmental correlation (*r*_E_ = 0.661, *p* < .001; Figure 3). No genetic or environmental correlations were found between either amygdala and the medial superior prefrontal cortex.

## 4 DISCUSSION

The aim of this study was to examine the genetic versus environmental contribution to cognitive and emotional brain function using both a multivariate ICA and a univariate ROI approach in twins. Our results suggested that heritability of brain function was specific to the task performed, with ICA‐derived working memory, sustained attention, nonconscious processing of positive and negative emotional faces, and selective attention brain function showing a small to moderate (21%–34%) genetic contribution, while no evidence of heritability was evident for the conscious processing of emotion and response inhibition using the ICA approach. When using an ROI approach, activation levels of the bilateral insula and medial superior prefrontal cortex showed similar levels of heritability (29%–34%) for the nonconscious processing of sad faces and selective attention, but not for the conscious processing of positive/negative emotion, working memory, sustained attention, and response inhibition processes. Lastly, our multivariate modelling results revealed both shared genetic and unique environmental correlations across the heritable brain networks and regions.

Although twin studies of task‐related fMRI relevant to emotion and cognitive processing are limited, the current multivariate findings (with heritability ranging from 21% to 34%) are somewhat consistent with previous univariate twin studies, together demonstrating small to moderate heritability for working memory‐related activation in the dorsolateral prefrontal cortex (Blokland et al., 2008, 2011) and the frontoparietal network (Etzel et al., 2020; Tang et al., 2021). This suggests that there is an overarching influence of genetics on working memory‐related brain function. Specifically, for working memory processes, 27% heritability was found for functional covariation in the frontoparietal network, which consisted of the right dorsolateral prefrontal cortex (dlPFC), inferior parietal lobule, and supramarginal gyrus. This is in line with previous studies showing heritability of these discrete frontoparietal regions involved in working memory and executive function (Blokland et al., 2008, 2011; Koten Jr et al., 2009; Matthews et al., 2007), and largely overlaps with the so‐called executive control network, strongly involved with top‐down attentional control and working memory processes (Fox et al., 2005; van den Heuvel & Hulshoff Pol, 2010; Vincent et al., 2008). Functional covariation among these regions indicates that this network has a measurable genetic basis in psychiatrically healthy adult twins, confirming and extending previous similar multivariate findings (Etzel et al., 2020).

Heritability for brain function associated with sustained attention was evident in two distinct networks. First, a posterior salience network comprising the superior temporal gyrus, precentral gyrus and the insula (Menon & Uddin, 2010) was observed, indicating moderate heritability (33%) for processes related to the detection of salient stimuli and initiation of attentional control (Menon & Uddin, 2010). A second network consisting of pre‐ and postcentral gyri and the inferior parietal lobule (IPL) also showed similar levels of heritability (34%), likely to be related to the co‐ordination of task representation and somatosensory processes. Taken together, our findings indicate a genetic basis for attentional processes that are subserved by these two networks working together to generate appropriate responses to salient stimuli, with the IPL working as an integration ‘hub’ and observed in tasks that probe general executive function (Igelström & Graziano, 2017; Tomasi & Volkow, 2011).

For the Oddball task, two networks related to selective attention showed moderate levels of heritability. One network consisting of the middle and superior temporal gyri showed heritability of 33% and is likely to be associated with the auditory processing of targets, which is known to have a significant genetic basis as indicated in previous studies (e.g., Babajani‐Feremi, 2017; Morell et al., 2007). In terms of task‐specific processes (i.e., related to the selection of the oddball/target stimuli from the nontarget stimuli, rather than general auditory processing across all stimuli), we found a network comprising the dlPFC, IPL, supramarginal gyrus, middle temporal gyrus, and angular gyrus to be moderately heritable (32%). These regions suggest the activation of the dorsal frontoparietal network, which is typically engaged during top‐down attentional processing of sensory inputs (Bareham et al., 2018; Melcher & Gruber, 2006), and relevant to our oddball task where participants were asked to selectively attend and respond to the oddball stimuli which require attentional control (Salmi et al., 2009; Wu et al., 2007). Intrinsic connectivity studies using resting state data have reported heritability estimates between 20% and 33% for a general attention network (Adhikari et al., 2018) and 33% for the dorsal attention network (Yang et al., 2016), which is similar to what we observed here. Furthermore, we also found significant heritability using the ROI method in the medial superior PFC (29%). This further strengthens the evidence for a genetic basis for the frontal regions associated with selective attention processes.

Contrary to our hypotheses, the only emotion‐related network that showed significant heritability was in the superior temporal gyrus and the insula when processing negative emotions unconsciously for Disgust > Neutral (26%) and Fear > Happy (23%) contrasts in the Nonconscious Faces task. The non‐specificity of the negative emotions across these contrasts suggests this network may be associated with an overall *negative* emotion processing compared to positive or neutral emotions; however, as this was not a hypothesis tested in the current study, this speculation will need to be explicitly tested in future studies (e.g., either by computing an overall negative minus positive/neutral contrast, or by using a conjunction approach; Friston et al., 2005). Results of the twin modelling suggest that genetics play a significant role when distinguishing between negative affect (especially related to disgust and fear) from others *on a subconscious level*, which may be an evolutionary adaptation where awareness of negative, threat‐related stimuli was key to survival (Flannelly et al., 2007), and may not be observed for positive affect. This is further bolstered by our ROI results of heritability for both the left (33%) and right (34%) amygdala in response to sad faces, indicating the possible significance of negative, compared to positive, emotional affect (although we note that we did not observe heritability effects for angry faces). Surprisingly, we did not find any networks that included amygdala function during the emotion task using the ICA approach, confirming the utility of employing both uni‐ and multivariate methods, as they may provide complimentary results that cannot be observed using a single method.

Interestingly, the components related to the conscious processing of facial expressions showed no evidence of a genetic basis using ICA or ROI approaches despite previous behavioural and EEG studies showing low to moderate heritability for conscious emotion processing (Anokhin et al., 2010; Routledge et al., 2018). However, these studies did not use an Emotion > Neutral contrast approach, and their results may therefore have included general face processing. We further speculate that the conscious processing of emotional stimuli may be more heavily influenced by environmental factors due to its importance in social functioning in daily life, where the intentional (conscious) and accurate perception of others' emotional expressions within a particular environmental context is a paramount skill for successful social interactions (de Paiva‐Silva et al., 2016; Walle & Lopez, 2020). This is also supported by studies that show differential (conscious) emotion recognition in adults with atypical development (e.g., having a history of childhood maltreatment), suggesting that environmental factors could be a stronger influence over and above genetics for conscious emotional processing (e.g., Bérubé et al., 2021).

Together, the heritability results indicate that functional networks related to executive functions (including attentional control, working memory, nonconscious emotion processing but not response inhibition/selection and conscious emotion processing) show genetic influences. Genetics also have a large impact on behavioural and psychological phenotypes (Bouchard et al., 2013; Johnson et al., 2009). However, research into brain development has shed light on how environmental factors may also shape individual differences, highlighting the importance of life experience on brain function via a feedback loop (i.e., epigenetics), or via direct impacts on the brain as one develops from childhood into adulthood (for a review, see Maggioni et al., 2020). Response inhibition and conscious awareness of facial emotions may be particularly sensitive to unique environmental factors as both processes are context‐dependent: response inhibition is tied to the selection of appropriate behaviour depending on the context (Mostofsky & Simmonds, 2008); and the conscious processing of facial expressions is crucial for successful personal interactions that are dependent on the (social) environmental context (Connolly et al., 2020). Therefore, the importance of these processes in successful social interactions within one's environment may lead them to be more susceptible to extrinsic influences.

Finally, our multivariate twin analyses revealed a pattern of both shared genetic and unique environmental covariance between networks/regions that showed significant heritability. For our ICA networks, there was evidence of shared genetic correlations between the Fear > Happy contrast and one of the sustained attention networks, as well as between the two sustained attention networks, and unique environmental correlations across the five components associated with cognitive functioning. The significant genetic correlations may indicate either pleiotropy where there is a shared genetic basis underlying the observed phenotype (the neural activity), or may also be due to assortative mating and gene–gene interactions, which were not accounted for in the current study (Hackinger & Zeggini, 2017). In terms of the environmental correlations, our results reveal an interesting pattern whereby the unique environmental covariance was observed across only the networks associated with cognitive functioning (e.g., sustained and selective attention, working memory), and not with facial expression emotion processing. It may be that the presence of external events specific (or unique) to each twin may play an essential role in the phenotypic expression of brain activity that underlie cognitive‐specific processes, rather than a common genetic basis. We speculate this may be related to the development of expertise (e.g., working in a job that requires a high level of executive functioning) during the lifespan that is particular for the individual, rather than being twin‐related (especially as our sample had a mean age of 39 years). For our ROIs, there was evidence of a very large shared genetic correlation between the activity elicited by the Sad > Neutral contrast in the bilateral amygdala. This is in line with morphology studies that suggest that the two hemispheres genetically mirror each other (Chen et al., 2011; Wen et al., 2016), and that there is a common genetic factor accounting for corresponding regions bilaterally (Eyler et al., 2014, which also found interhemispheric genetic correlations that were statistically indistinguishable from being perfectly correlated; *r* = 1.0).

Several strengths and limitations of our study are worth noting. The current study is one of the first using a data‐driven multivariate ICA method to characterise the heritability of task‐related functional networks (but see Etzel et al., 2020; Tang et al., 2021)^1^ from a relatively large fMRI sample of healthy community adults, and the first to examine shared genetic and environmental correlations across heritable brain networks/regions spanning multiple tasks, to the best of our knowledge. Yet when we consider ROI approaches, the smaller number of task‐based fMRI studies previously conducted in twin research (compared to structural or resting state MRI) highlights the difficulties of measuring and comparing heritability on group‐level (i.e., averaged) brain activation due to individual variability and researcher bias (e.g., selecting regressors, modelling the design matrix, choosing regions‐of‐interest). In fact, group‐level activation and heritability may only show partial overlap as functional *variability* is more likely to be affected by genetic effects rather than an overall averaged pattern of activation (Koten Jr et al., 2009); however, we note that such individual variability may also place an upper limit on the heritability estimates due to a greater impact of unique environment factors. Furthermore, using contrasts between conditions may have led to reduced heritability effects, as calculating a difference score may remove some of the genetic variance (Li et al., 2019), especially in the ROI analyses. This is worth noting here as a potential limitation of this study as it is possible that our ROI analyses did not reliably reveal the true heritability of each region based on these limitations. Further, due to equipment failure, behavioural performance was not included as a covariate across all five tasks in order to keep the MRI analyses consistent, which could have potential task performance effects on brain activity. Future studies should consider the inclusion of behavioural data when evaluating task‐relevant fMRI signal. Standard limitations regarding the use of twin models also needs to be mentioned, including assumptions of equal environments and random mating, and a lack of generalisability of results across different sample populations. The unique environment factor (*E*) also includes measurement error, which places an upper limit on heritability estimates. This limit is due to measurement‐specific variance being removed from the pool of variance that is due to genetics (Blokland et al., 2012). Lastly, there has been much debate regarding the test–retest reliability of fMRI task‐based activation, with findings suggesting that basic (vs. complex) tasks that examine cortical (vs. subcortical) activations in nonclinical (vs. clinical) populations exhibit higher reliability (Bennett & Miller, 2010; Noble et al., 2021). On this note, multivariate methods have been shown to result in greater reliability compared to univariate models (Noble et al., 2021), which may partially explain the potential lack of heritability observed in our ROI analyses. This will require further investigation in an independent sample.

In conclusion, we provide novel evidence for the heritability of functional brain networks engaged during cognitive and emotion tasks using two methodological approaches, bridging a large gap between those studies solely focusing on resting state connectivity (task‐free) and those studies utilising a task‐based general linear model approach (univariate) alone. By examining multiple tasks within the same group of psychiatrically healthy adult twins, we observed differential patterns of genetic and environmental influences for these task‐related networks, with brain function related to attention, working memory, and nonconscious processing of negative affect showing a genetic influence, while response inhibition and conscious processing of emotion being driven more by unique environmental factors. We further show the benefit of using a multivariate ICA approach (which decomposes whole‐brain signal into independent networks) in addition to the univariate ROI approach (which uses *a priori* anatomical regions based on hypotheses) as ICA is able to include a wider range of regions into the analysis by examining covarying patterns of brain function, rather than focusing only on discrete regions. The influence of genetics and environment are differentially distributed across functional domains, indicating a complex relationship between genetic factors and environmental circumstances. Such complexity is further supported by the evidence of both common genetic and unique environmental pathways across different heritable networks and regions, and in particular across cognitive processes, which seem to largely be driven by unique environmental sources, such as life experiences that are not shared between twins. Future work examining both structural and functional connectivity simultaneously may reveal additional insights into the effect of genetics and environment on neural phenotypes that lead to individual differences.

## AUTHOR CONTRIBUTIONS

**Haeme R. P. Park:** Conceptualization, Methodology, Software, Formal analysis, Writing – original draft, Writing – review & editing, Visualization. **Miranda R. Chilver:** Data Curation, Writing – review & editing. **Yann Quidé:** Methodology, Writing – review & editing. **Arthur Montalto:** Software, Writing – review & editing. **Peter R. Schofield:** Conceptualization, Resources, Writing – review & editing, Project administration, Funding acquisition. **Leanne M. Williams:** Conceptualization, Resources, Writing – review & editing, Project administration, Funding acquisition. **Justine M. Gatt:** Conceptualization, Investigation, Resources, Writing – review & editing, Supervision, Project administration, Funding acquisition.

## CONFLICT OF INTEREST STATEMENT

Leanne M. Williams has received advisory board fees from One Mind Psyberguide and the Laureate Institute for Brain Research unrelated to this study. Justine M. Gatt is a stockholder in MAP Biotech Pty Ltd. All other authors declare that they have no conflicts of interest.

## Supporting information


**Appendix S1:** Supporting Information

## Data Availability

The data that support the findings of this study are available on request from the corresponding author. The data are not publicly available due to privacy or ethical restrictions.

## Footnotes

^1^
Both these studies examined genetic contributions of cognitive control using multivariate pattern similarity analyses on task‐based fMRI data; however, parcellation methods were applied to delineate regions of interest, in contrast to the whole‐brain approach used in the current study.
