# Supplementary material for: Heritability of cognitive and emotion processing during functional MRI in a twin sample
Source: Hum Brain Mapp. 2024 Jan 12;45(1):e26557. doi: 10.1002/hbm.26557 (PMC10785190; doi:10.1002/hbm.26557)
Supplement: Supplementary file 1 — Appendix S1: Supporting Information [file HBM-45-e26557-s001.docx]

**Supplementary Materials for**

**Heritability of cognitive and emotion processing during functional MRI in a twin sample**

Haeme R.P. Park^1,2^, Miranda R. Chilver^1,2^, Yann Quidé^1,2^, Arthur Montalto^1,2^, Peter R. Schofield^1,3^, Leanne M. Williams^4^, Justine M. Gatt^1,2^

^1^ Neuroscience Research Australia, Sydney, NSW, Australia

^2^ School of Psychology, University of New South Wales, Sydney, NSW, Australia

^3^ School of Biomedical Sciences, University of New South Wales, Sydney, NSW, Australia

^4^ Psychiatry and Behavioral Sciences, Stanford School of Medicine, Stanford University, Stanford, California, USA

This document contains:

S1. Supplementary Methods and Materials

- S1.1 fMRI paradigms
- S1.2 Image acquisition and preprocessing
- S1.3 Independent component analyses
- S1.4 Regions-of-interest selection
- S1.5 Regions-of-interest analyses

S2. Supplementary Results

- S2.1 ICA results
  - S2.1.1 – S2.1.5 ICA tables
- S2.2 Intra-class correlations between MZ and DZ twins
- S2.3 Number of twins included in fMRI and twin modelling analyses

**Corresponding author:**

Justine Gatt

Neuroscience Research Australia

Margarete Ainsworth Building

Barker Street, Randwick 2031

Australia

Email: j.gatt@unsw.edu.au

Phone: +61 2 9399 1812

**S1 Methods and Materials**

*S1.1 fMRI paradigms*

In brief, both the nonconscious and conscious conditions of the Facial Expressions of Emotion Test (FEET) consisted of 240 standardised faces depicting anger, fear, sadness, disgust, happiness, or neutral expressions, which were presented in a blocked design. Eight different faces of the same emotional expression were presented as one block, before moving onto another emotion. Five blocks were presented during each task for each emotion (30 blocks in total per task). For the Nonconscious version, the emotional face stimulus was presented for 10 ms before being masked by a neutral face for 190 ms in order to prevent conscious processing of the emotion, which was then followed by an interstimulus interval (ISI) of 1050 ms. For the Conscious version, the emotional face stimulus was presented for 500 ms, followed by an ISI of 750 ms. Both facial expressions within each block and different emotions between blocks were pseudorandomised. As it was a passive viewing paradigm, participants were not required to respond; however, instructions were given to pay close attention to the faces and that follow-up questions may be asked. After the completion of both Nonconscious and Conscious paradigms, each participant was probed on the number of different emotions they observed for each task.

In the N-back task, 120 stimuli (B, C, D, or G letters) were presented serially, with each letter being on screen for 200 ms, followed by an ISI of 2300 ms. 50 of the letters were in yellow to be held in working memory (no consecutive repetition of the same letter), while 30 were 1-back sustained attention stimuli (consecutive repetition) that required participant responses. 40 letters were presented in white and were perceptual baseline stimuli. Reaction time and accuracy were measured with participants scoring 2 standard deviations above the mean accuracy being removed from further analyses.

For the Go-NoGo task, participants were presented with 180 Go and NoGo stimuli, which were the word ‘press’ in green and red, respectively. Participants were asked to respond to the Go stimuli, but withhold (inhibit) responses to the NoGo stimuli. Each stimulus was presented for 500 ms with an ISI of 750 ms. NoGo stimuli were never presented more than three times consecutively. Reaction time and accuracy were measured and used to evaluate successful inhibition task performance during the task.

Finally, the Oddball task consisted of 20 target tones presented at 1000 Hz, and 100 nontarget tones presented at 50 Hz for 50 ms each in a serial manner, followed by an ISI of 2400 ms after each tone. Participants were asked to respond to the target tones only. The decibel level was set at 75 dB for all tones.

*S1.2 Image acquisition and preprocessing*

MR images were acquired on a 3T GE Signa HDx scanner (GE Healthcare, Milwaukee, WI) at Westmead Hospital Medical Imaging Service in Sydney, using an eight-channel head coil. 3D T1-weighted volumes were first collected using a spoiled gradient echo (SPGR) sequence with the following parameters: TR= 8.3ms; TE= 3.2ms; flip angle = 11 degrees; inversion time = 500ms; FOV= 256mm; 180 sagittal slices; matrix size =256 ×256; voxel size =1× 1×1mm; NEX=1; ASSET=1.5; scanning time = 7.12 minutes. The five functional runs all consisted of 120 volumes of T2*-weighted echo-planar images (EPI) with the following parameters: TR = 2500 ms; TE = 27.5 ms; FOV= 240mm; flip angle = 90 degrees; 40 axial slices using an interleaved sequence; matrix size = 64 × 64; voxel size = 3.75 × 3.75 × 3.5 mm; scanning time = 5.13 minutes for each run.

Each of the five tasks were analysed separately using SPM12 software (Wellcome Trust Centre for Neuroimaging, London, UK) implemented in Matlab 2018b (MathWorks, Natick, MA, USA). For all tasks, the first three EPI volumes were discarded to allow for tissue magnetisation to reach equilibrium. Both the remaining functional and structural images were coregistered to the SPM template. The structural image was then segmented then spatially normalised to the standard Montreal Neurological Institute (MNI) space, while the functional EPIs were slice-time corrected, realigned, then normalised to MNI space using the deformation fields generated from the structural image, then resliced to a voxel size of 1.5 × 1.5 × 2 mm, and smoothed with an isotropic Gaussian filter of 8 × 8 × 8 mm at full-width half-maximum.

*S1.3 Independent component analyses*

In ICA, a multivariate signal is decomposed into statistically independent components. During this process, a principal components analysis is first run in order to reduce each dataset, which is then followed by an ICA decomposition. For this, we used the Infomax algorithm to separate each dataset into a number of maximally independent components (IC), which was first estimated using the minimum description length criteria for each task. The algorithm was repeated 20 times using ICASSO for reliability and consistency of the generated components. Finally, spatial maps and associated time courses for each participant were back-reconstructed, and averaged spatial maps for each component per task were thresholded at z = 3.5 to identify the brain regions within the component. Regions with high z-scores within each component reflect larger contribution to the time course, and can be taken as a map of functional connectivity, due to the regions all sharing a single time course. The quality of the ICs was assessed using the ICASSO cluster quality index (Iq), where any components with an Iq value of less than 0.9 were discarded. In addition, the ICs were spatially correlated with white matter and cerebrospinal fluid tissue probability maps from SPM12, and those with an *r* coefficient of more than 0.25 were also discarded.

*S1.4 Regions-of-interest selection*

The ROIs used in the current study were selected from a previous study that quantified 6 brain circuits related to emotional and cognitive functions observed in mental health symptoms (depression and anxiety; Goldstein-Piekarski et al., 2022). In more detail, the meta-analytic database ‘Neurosynth.org’ was used to generate 6 target circuits-of-interest, which included the default mode, salience, attention, negative affect, positive affect, and cognitive control circuits. The regions within these circuits were then refined by excluding areas with low temporal signal-to-noise ratio and low fit to grey matter. We then chose the ROIs corresponding to task-related circuits (attention, negative and positive affect, and cognitive control) that were probed by our 5 tasks (N-back, Oddball, Nonconscious and Conscious Facial Expressions of Emotion, and Go-NoGo).

*S1.5 Regions-of-interest analyses*

For both the nonconscious and conscious conditions of the Facial Expressions of Emotion Test, the time courses modelled from convolving a boxcar stimulus function with a haemodynamic response function was applied to the general linear model, which included each of the 6 experimental regressors (Happy, Angry, Sad, Fear, Disgust, and Neutral), as well as 6 movement regressors (of no interest that were derived from the realignment step). For the cognitive tasks, BOLD responses were modelled using a delta stick function, with the N-back task matrix consisting of 3 experimental regressors (Target, NonTarget, Baseline) and 6 movement regressors, the Go-NoGo task matrix consisting of two experimental (Correct Go and Correct NoGo) and 6 movement regressors, and the Oddball task matrix also consisting of two experimental (Target, NonTarget) and 6 movement regressors.

**S2 Results**

*S2.1 ICA results*

*Nonconscious FEET:* 27 ICs were identified for the Nonconscious Faces task. By conducting targeted *t*-tests with the beta weights for each condition (Angry, Fear, Disgust, Sad, Neutral, and Happy), we identified two components that were associated with emotion processing. This included: IC4, consisting of the superior temporal gyrus and insula, which was associated with Angry > Neutral, Disgust > Neutral, and Fear > Happy contrasts; and IC11, consisting of the cuneus and lingual gyrus, and associated with Angry > Happy, Fear > Happy, and Neutral > Happy contrasts.

*Conscious FEET:* 27 ICs were identified for the Conscious Faces task. From the *t*-tests, nine components were found to be related to emotion-related contrasts. In brief, IC2 consisted of the lingual gyrus and cuneus, and was associated with all emotion-neutral contrasts (i.e., Angry > Neutral, Happy > Neutral, Fear > Neutral, Disgust > Neutral, and Sad > Neutral), while IC5 consisted of the precuneus, cuneus and the posterior cingulate cortex, and was associated specifically with the Disgust > Neutral contrast. IC6 included the superior temporal gyrus and the insula, and was associated with Angry > Neutral, Happy > Neutral, Disgust > Neutral, Fear > Happy, and Sad > Happy contrasts. Both IC11 and IC15 were related to negative emotion > Happy contrasts, with IC11 consisting of a frontal-parieto-temporal network for Fear> Happy and Disgust > Happy contrasts, and IC15 consisting of pre- and postcentral gyri for the Angry > Happy contrast. IC9 and IC17 were related to negative emotion-neutral contrasts, with IC9 related to Angry > Neutral and Fear > Neutral, and consisting of occipital activation, while IC17 showed covariation in the inferior and middle frontal gyri for Fear > Neutral, Disgust > Neutral, and Sad > Neutral contrasts. IC16 included lingual gyrus, culmen, and the posterior cingulate cortex, for Happy > Neutral and Disgust-Neutral contrasts. Finally, IC23 was related to fearful faces (Fear > Neutral, Fear > Happy) in the middle and superior temporal gyri.

*N-back:* 26 ICs were identified for the CPT task. When beta weights for each condition (Target, NonTarget, Baseline) were subjected to a one-way ANOVA, four ICs were found to be significantly associated with the task (IC4, IC10, IC12, and IC14). Two post-hoc contrasts were run to examine which of these four components were specifically related to sustained attention (Target > Baseline) and working memory (NonTarget > Baseline) processes. IC4, IC10, and IC14 were associated with the sustained attention contrast, while IC4, IC12, and IC14 were also associated with the working memory contrast. IC4 consisted of superior temporal gyrus, precentral gyrus, and the insula, while IC10 included precentral and postcentral gyri, as well as the inferior parietal lobule. IC12 consisted of a right lateralised fronto-parietal network, including activations in the inferior and superior frontal gyri, inferior parietal lobule, and the supramarginal gyrus. IC14 included left lateralised functional connections between fronto-parieto-temporal regions, including the inferior and medial frontal gyri, precuneus, angular and supramarginal gyri, and the inferior parietal lobule.

*Oddball:* 27 ICs were identified for the Oddball task. After excluding eight components that failed quality control (spatial *r* > 0.20; Iq < 0.90), nine components were found to be associated with novelty detection using the Target > NonTarget contrast. These included activations in the occipital and posterior parietal regions (IC3, IC12), frontal regions including the insula (IC9, IC16), fronto-parietal (IC7) and fronto-temporal (IC6) networks, basal ganglia (IC5), superior and middle temporal gyri (IC11), and a fronto-parieto-temporal network including the middle and superior frontal gyri, angular and supramarginal gyri, middle temporal gyrus, and the inferior parietal lobule (IC23).

*Go-NoGo:* 22 ICs were identified for the Go-NoGo task with one IC being excluded due to having an Iq less than 0.90. From the one-way ANOVA, we identified three components that were specifically related to successful inhibition (CorrectNoGo > CorrectGo) by running a post-hoc contrast. These included: IC3 comprising of cerebellum regions; IC7 comprising of the precuneus; and IC14, which included the posterior cingulate cortex. One component was found to be specifically related to failed inhibition (IC10; FailedNoGo > CorrectNoGo), which comprised of superior temporal gyrus activation. Lastly, five other components were found to be associated with both successful and failed inhibition, potentially denoting general task-related activation. These included IC6 (temporal gyri and supramarginal gyrus), IC9 (temporo-parietal), IC12 (precuneus), IC15 (inferior parietal lobule), and IC16 (inferior parietal lobule extending to postcentral gyrus.

| Table S2.1.1 Independent components determined by minimum description length criteria for the nonconscious condition of the Facial Expressions of Emotion Test (FEET). | | | | | |
| --- | --- | --- | --- | --- | --- |
| Component | Voxel directionality | Brain region labels | Brodmann area | L/R volumes (cm^3^) | L/R: max z-value (x,y,z) |
| IC1 | Positive | Middle Occipital Gyrus | 19, 37 | 3.0/2.0 | 6.9 (-27, -79, 11)/6.2 (31, -75, 13) |
|  |  | Cuneus | 17, 18 | 1.5/0.4 | 6.2 (-22, -81, 11)/4.9 (28, -72, 13) |
|  |  | Sub-Gyral | - | 1.5/2.1 | 5.7 (-25, -76, 22)/5.9 (30, -73, 20) |
|  |  | Precuneus | 7, 31 | 0.6/1.0 | 5.2 (-24, -73, 18)/4.9 (27, -76, 20) |
|  |  |  |  |  |  |
| IC2 | Positive | Culmen | - | 3.1/2.9 | 13.5 (-6, -38, -8)/14.1 (6, -39, -6) |
|  |  |  |  |  |  |
| IC3 | Positive | Superior Temporal Gyrus | 21, 22, 38 | 2.0/2.2 | 13.4 (-40, 4, -15)/13.7 (40, 7, -15) |
|  |  | Sub-Gyral | 13, 21 | 2.1/1.2 | 11.2 (-42, 0, -10)/10.7 (43, 1, -10) |
|  |  | Inferior Frontal Gyrus | 13, 47 | 1.2/1.0 | 10.3 (-39, 11, -14)/9.6 (36, 14, -16) |
|  |  |  |  |  |  |
| IC4 | Positive | Superior Temporal Gyrus | 13, 21, 22, 41, 42 | 6.3/4.9 | 7.3 (-53, -22, 7)/6.5 (55, -15, 6) |
|  |  | Insula | 13, 22 | 2.0/1.8 | 6.3 (-45, -17, 5)/5.4 (43, -14, 3) |
|  |  |  |  |  |  |
| IC5 | Positive | Extra-Nuclear | - | 1.8/1.4 | 8.2 (-3, -24, 7)/7.3 (6, -27, 7) |
|  |  | Thalamus | - | 2.4/2.3 | 6.8 (-4, -19, 8)/8.0 (4, -22, 8) |
|  |  |  |  |  |  |
| IC6 | Positive | Paracentral Lobule | 4, 5, 6, 31 | 3.1/2.3 | 7.9 (-3, -28, 64)/7.1 (4, -33, 62) |
|  |  | Medial Frontal Gyrus | 6 | 1.7/1.5 | 7.2 (-4, -24, 64)/7.4 (4, -27, 64) |
|  |  | Postcentral Gyrus | 2, 3, 4, 5, 40 | 1.9/1.3 | 6.9 (-4, -39, 63)/4.8 (22, -28, 62) |
|  |  | Precuneus | 7 | 1.1/* | 6.6 (-1, -48, 58)/5.3 (6, -49, 58) |
|  |  | Precentral Gyrus | 3, 4, 6 | 1.1/1.3 | 5.2 (-12, -31, 64)/5.2 (10, -27, 64) |
|  |  |  |  |  |  |
| IC7 | Positive | Medial Frontal Gyrus | 6, 8, 32 | 1.0/* | 5.9 (0, 10, 46)/* |
|  |  | Insula | 13, 47 | 1.8/* | 5.8 (-43, 13, -2)/* |
|  |  | Inferior Frontal Gyrus | 47 | */1.0 | */5.5 (45, 15, -2) |
|  |  | Cingulate Gyrus | 24, 32 | 1.4/1.3 | 5.2 (0, 21, 32)/5.2 (4, 13, 42) |
|  |  |  |  |  |  |
| IC8 | Positive | Inferior Parietal Lobule | 39, 40 | */2.4 | */7.3 (45, -59, 38) |
|  |  | Angular Gyrus | 39 | */1.2 | */7.1 (45, -60, 34) |
|  |  | Supramarginal Gyrus | 40 | */2.6 | */7.0 (50, -53, 34) |
|  |  | Middle Frontal Gyrus | 6, 8, 9, 10, 46 | */5.6 | */5.1 (30, 58, 6) |
|  |  | Superior Frontal Gyrus | 6, 8, 10 | */1.7 | */5.0 (28, 20, 49) |
|  |  |  |  |  |  |
| IC9 | Positive | Medial Frontal Gyrus | 6, 8, 9 | 2.3/1.8 | 6.6 (-1, 50, 34)/5.9 (3, 49, 34) |
|  |  | Superior Frontal Gyrus | 9 | 1.6/* | 6.2 (-1, 53, 31)/* |
|  |  |  |  |  |  |
| IC10 | Positive | Anterior Cingulate | 10, 24, 32 | 2.1/2.3 | 8.0 (-4, 44, 0)/8.7 (4, 41, 2) |
|  |  | Medial Frontal Gyrus | 10 | 2.0/1.5 | 7.9 (-1, 53, -4)/8.2 (3, 51, -4) |
|  |  |  |  |  |  |
| IC11 | Positive | Lingual Gyrus | 18, 19 | 4.0/3.8 | 11.0 (-1, -77, 0)/11.3 (3, -77, 0) |
|  |  | Cuneus | 17, 18, 23, 30 | 2.0/2.5 | 8.7 (-1, -79, 8)/9.8 (7, -75, 6) |
|  |  |  |  |  |  |
| IC12 | Positive | Precuneus | 7, 31 | 6.0/4.9 | 11.8 (0, -64, 46)/10.4 (4, -66, 46) |
|  |  |  |  |  |  |
| IC13 |  | None |  |  |  |
|  |  |  |  |  |  |
| IC14 | Positive | Angular Gyrus | 39 | 1.2/* | 9.2 (-43, -65, 35)/* |
|  |  | Middle Temporal Gyrus | 19, 39 | 3.2/* | 8.8 (-48, -64, 27)/* |
|  |  | Inferior Parietal Lobule | 7, 39, 40 | 2.0/* | 8.7 (-42, -63, 38)/* |
|  |  | Superior Temporal Gyrus | 22, 39 | 1.5/* | 8.4 (-49, -59, 27)/* |
|  |  | Supramarginal Gyrus | 40 | 1.9/* | 8.2 (-49, -56, 30)/* |
|  |  | Middle Frontal Gyrus | 6, 8 | 1.4/* | 4.7 (-31, 19, 47)/* |
|  |  |  |  |  |  |
| IC15 | Positive | Lentiform Nucleus | - | 1.5/1.8 | 4.4 (-18, 6, -4)/4.3 (21, 6, -4) |
|  |  |  |  |  |  |
| IC16 | Positive | Lingual Gyrus | 18, 19 | 1.3/1.0 | 8.7 (-9, -58, 5)/7.8 (10, -55, 5) |
|  |  | Culmen | 19 | 1.3/* | 8.3 (-9, -55, 1)/* |
|  |  | Posterior Cingulate | 23, 29, 30, 31 | 2.9/2.8 | 8.2 (-4, -57, 7)/7.6 (12, -56, 8) |
|  |  |  |  |  |  |
| IC17 | Positive | Inferior Parietal Lobule | 7, 39, 40 | 4.6/4.5 | 5.9 (-46, -41, 44)/5.8 (45, -45, 45) |
|  |  |  |  |  |  |
| IC18 | Positive | Precentral Gyrus | 3, 4, 6, 43 | 3.2/4.0 | 8.0 (-52, -11, 26)/7.8 (55, -6, 22) |
|  |  | Postcentral Gyrus | 2, 3, 43 | 3.1/1.7 | 7.7 (-53, -10, 23)/6.4 (56, -10, 23) |
|  |  |  |  |  |  |
| IC19 | Positive | - | - | 1.2/0.6 | 15.3 (0, -46, -25)/12.1 (3, -43, -25) |
|  |  | Nodule | - | */1.0 | */15.9 (0, -48, -28) |
|  |  | Culmen | - | 2.2/1.8 | 10.9 (-3, -47, -21)/11.6 (3, -47, -21) |
|  |  |  |  |  |  |
| IC20 | Positive | Medial Frontal Gyrus | 6, 9, 10 | 3.5/3.3 | 7.8 (-4, 58, 17)/7.5 (4, 58, 16) |
|  |  | Superior Frontal Gyrus | 9, 10 | 1.0/1.0 | 7.0 (-3, 57, 25)/7.0 (3, 57, 25) |
|  |  |  |  |  |  |
| IC21 | Positive | Middle Frontal Gyrus | 9, 10, 46 | 2.6/1.0 | 7.5 (-45, 26, 19)/5.1 (45, 32, 17) |
|  |  | Sub-Gyral | - | 3.6/* | 7.4 (-46, 22, 21)/* |
|  |  | Inferior Frontal Gyrus | 9, 45, 46 | 3.7/1.1 | 7.0 (-43, 33, 13)/4.9 (45, 35, 13) |
|  |  | Precuneus | 19, 39 | 1.0/* | 4.8 (-31, -66, 35)/* |
|  |  |  |  |  |  |
| IC22 | Positive | Postcentral Gyrus | 40 | */1.0 | */5.3 (56, -25, 16) |
|  |  | Superior Temporal Gyrus | 22, 41, 42 | */1.0 | */4.8 (61, -26, 16) |
|  |  |  |  |  |  |
| IC23 | Positive | Precuneus | 7, 23, 31 | 3.8/3.5 | 9.9 (-1, -69, 29)/9.3 (3, -69, 29) |
|  |  | Cuneus | 7, 18, 19 | 1.1/1.0 | 8.4 (-3, -72, 33)/8.1 (3, -72, 33) |
|  |  | Cingulate Gyrus | 23, 31 | 1.1/* | 5.7 (-1, -58, 29)/* |
|  |  | Posterior Cingulate | 23, 29, 30, 31 | 1.5/1.3 | 6.2 (0, -46, 23)/5.9 (4, -41, 20) |
|  |  |  |  |  |  |
| IC24 | Positive | Inferior Frontal Gyrus | 44, 45, 47 | 3.5/* | 6.9 (-49, 18, -3)/* |
|  |  | Superior Temporal Gyrus | 13, 22, 39 | 2.8/1.2 | 6.2 (-52, -57, 14)/4.4 (55, -50, 14) |
|  |  | Middle Temporal Gyrus | 19, 21, 37, 39 | 3.1/* | 5.8 (-53, -56, 10)/* |
|  |  |  |  |  |  |
| IC25 | Positive | Inferior Parietal Lobule | 40 | 1.1/* | 5.0 (-56, -35, 22)/* |
|  |  |  |  |  |  |
| IC26 | Positive | Declive | - | 6.0/5.5 | 7.3 (-28, -69, -20)/7.0 (31, -69, -20) |
|  |  |  |  |  |  |
| IC27 | Positive | Cuneus | 7, 17, 18, 19 | 4.5/4.0 | 10.2 (-3, -77, 26)/10.4 (1, -77, 28) |
|  |  | Precuneus | 7, 31 | 1.3/1.8 | 8.2 (-6, -74, 28)/8.4 (1, -73, 26) |
|  |  |  |  |  |  |
| Note: L/R: left/right; *: non-significant voxels or voxels with *z<*3.5 and volume smaller than 1cm^3^ | | | | | |
|  | | | | | |
| Table S2.1.2. Independent components determined by minimum description length criteria for the conscious condition of the FEET. | | | | | |
| Component | Voxel directionality | Brain region labels | Brodmann area | L/R volumes (cm^3^) | L/R: max z-value (x,y,z) |
| IC1 | Positive | Culmen | - | 2.9/2.8 | 14.1 (-6, -39, -8)/13.2 (7, -39, -8) |
|  |  |  |  |  |  |
| IC2 | Positive | Lingual Gyrus | 18, 19 | 3.2/3.4 | 11.5 (-1, -78, 1)/12.1 (3, -78, 1) |
|  |  | Cuneus | 17, 18, 23, 30 | 2.0/2.3 | 9.2 (-1, -78, 8)/10.1 (3, -78, 8) |
|  |  |  |  |  |  |
| IC3 | Positive | Superior Temporal Gyrus | 22, 38 | 2.3/1.6 | 14.2 (-40, 4, -15)/14.7 (40, 7, -15) |
|  |  | Inferior Frontal Gyrus | 13, 47 | 1.1/* | 12.2 (-34, 10, -17)/* |
|  |  | Sub-Gyral | 21 | 2.0/1.4 | 11.1 (-43, -1, -10)/11.4 (43, -2, -12) |
|  |  |  |  |  |  |
| IC4 | Positive | Paracentral Lobule | 4, 5, 6, 31 | 3.2/2.4 | 8.5 (-1, -42, 61)/8.3 (3, -28, 64) |
|  |  | Postcentral Gyrus | 2, 3, 4, 40 | 1.6/1.1 | 7.6 (-4, -39, 63)/5.2 (13, -33, 64) |
|  |  | Medial Frontal Gyrus | 4, 6 | 1.7/1.4 | 7.5 (-4, -24, 64)/7.5 (4, -24, 64) |
|  |  | Precuneus | 7 | 1.4/* | 6.4 (-4, -49, 58)/* |
|  |  | Sub-Gyral | 40 | 1.1/* | 5.8 (-9, -40, 61)/* |
|  |  | Precentral Gyrus | 4 | 1.0/* | 5.6 (-9, -24, 64)/* |
|  |  |  |  |  |  |
| IC5 | Positive | Precuneus | 7, 23, 31 | 3.3/3.3 | 10.4 (-1, -70, 27)/9.6 (3, -70, 27) |
|  |  | Cuneus | 7, 18, 19 | 1.5/1.0 | 8.8 (-3, -68, 31)/8.7 (4, -71, 31) |
|  |  | Posterior Cingulate | 23, 29, 30, 31 | 1.7/1.5 | 6.2 (0, -45, 23)/6.0 (4, -42, 19) |
|  |  |  |  |  |  |
| IC6 | Positive | Superior Temporal Gyrus | 13, 21, 22, 41 | 6.5/5.2 | 7.0 (-50, -24, 7)/6.7 (53, -17, 6) |
|  |  | Insula | 13, 22 | 2.0/1.9 | 6.1 (-45, -14, 3)/6.4 (46, -8, 0) |
|  |  |  |  |  |  |
| IC7 | Positive | Inferior Parietal Lobule | 7, 40 | 4.5/2.6 | 6.0 (-40, -47, 48)/5.2 (43, -42, 48) |
|  |  | Precuneus | 7, 19 | 3.1/2.8 | 5.7 (-19, -67, 46)/4.9 (16, -69, 44) |
|  |  | Superior Parietal Lobule | 7, 40 | 1.0/* | 5.3 (-22, -64, 47)/* |
|  |  |  |  |  |  |
| IC8 | Positive | Anterior Cingulate | 10, 24, 32 | 1.7/2.0 | 8.9 (-3, 40, 4)/11.1 (1, 40, 4) |
|  |  | Medial Frontal Gyrus | 10 | 1.6/1.7 | 7.8 (-1, 49, 3)/6.5 (3, 56, 1) |
|  |  |  |  |  |  |
| IC9 | Positive | Declive | - | 4.4/3.5 | 9.0 (-31, -72, -18)/7.7 (36, -69, -18) |
|  |  | Middle Occipital Gyrus | - | 1.2/1.2 | 6.7 (-27, -83, 1)/5.7 (31, -80, 2) |
|  |  | Sub-Gyral | - | */1.0 | */6.0 (31, -81, -1) |
|  |  |  |  |  |  |
| IC10 | Positive | Cuneus | 7, 17, 18, 19, 23 | 4.7/4.5 | 9.7 (-1, -79, 21)/10.5 (3, -79, 19) |
|  |  | Precuneus | 7, 31 | 1.2/1.6 | 8.3 (-1, -74, 26)/7.4 (1, -72, 29) |
|  |  |  |  |  |  |
| IC11 | Positive | Inferior Parietal Lobule | 7, 40 | */2.5 | */7.2 (43, -60, 38) |
|  |  | Angular Gyrus | 39 | */1.2 | */6.9 (46, -57, 36) |
|  |  | Supramarginal Gyrus | 40 | */2.4 | */6.1 (50, -50, 36) |
|  |  | Middle Frontal Gyrus | 8, 9, 10, 46 | */4.9 | */5.4 (31, 58, 4) |
|  |  | Middle Temporal Gyrus | 39 | */1.0 | */5.2 (45, -64, 27) |
|  |  |  |  |  |  |
| IC12 | Positive | Nodule | - | 0.8/1.0 | 10.3 (-3, -50, -28)/12.1 (0, -48, -28) |
|  |  | - | - | 1.8/1.7 | 9.8 (-3, -45, -26)/10.0 (3, -44, -25) |
|  |  | Culmen | - | 1.7/1.9 | 8.7 (-3, -47, -21)/9.0 (3, -47, -21) |
|  |  | Uvula | - | 1.0/1.0 | 5.5 (-1, -64, -27)/5.7 (1, -61, -27) |
|  |  | Declive | - | 1.4/2.0 | 5.2 (-4, -65, -20)/5.1 (4, -65, -20) |
|  |  | Pyramis | - | 1.0/1.0 | 5.0 (-6, -65, -24)/5.0 (4, -66, -24) |
|  |  |  |  |  |  |
| IC13 | Positive | Precuneus | 7 | 2.2/1.9 | 11.4 (-1, -52, 56)/10.1 (3, -64, 46) |
|  |  |  |  |  |  |
| IC14 | Positive | Angular Gyrus | 39 | 1.1/* | 9.5 (-49, -58, 34)/* |
|  |  | Supramarginal Gyrus | 40 | 2.5/* | 9.3 (-50, -56, 30)/* |
|  |  | Inferior Parietal Lobule | 39, 40 | 1.6/* | 9.0 (-48, -57, 38)/* |
|  |  | Middle Temporal Gyrus | 19, 39 | 2.8/* | 8.9 (-49, -61, 29)/* |
|  |  | Superior Temporal Gyrus | 22, 39 | 1.5/* | 8.5 (-50, -56, 27)/* |
|  |  |  |  |  |  |
| IC15 | Positive | Postcentral Gyrus | 2, 3, 43 | 3.1/1.6 | 7.5 (-53, -10, 24)/6.4 (59, -5, 15) |
|  |  | Precentral Gyrus | 3, 4, 6, 43 | 3.1/4.2 | 7.4 (-52, -11, 28)/7.4 (56, -4, 22) |
|  |  |  |  |  |  |
| IC16 | Positive | Culmen | 19 | 2.6/1.9 | 9.0 (-9, -53, -1)/7.6 (10, -51, -1) |
|  |  | Lingual Gyrus | 18, 19 | 2.1/1.8 | 8.1 (-10, -56, 3)/7.3 (13, -53, 1) |
|  |  | Posterior Cingulate | 29, 30, 31 | 1.9/1.9 | 6.3 (-15, -59, 7)/6.2 (13, -56, 8) |
|  |  |  |  |  |  |
| IC17 | Positive | Sub-Gyral | - | 3.1/* | 7.4 (-46, 22, 21)/* |
|  |  | Inferior Frontal Gyrus | 6, 9, 44, 45, 46 | 5.1/1.2 | 7.3 (-45, 28, 17)/4.6 (50, 29, 17) |
|  |  | Middle Frontal Gyrus | 9, 10, 46 | 2.2/* | 7.2 (-45, 32, 15)/* |
|  |  |  |  |  |  |
| IC18 | Positive | Extra-Nuclear | - | 1.8/1.0 | 8.3 (-3, -25, 7)/7.9 (4, -30, 7) |
|  |  | Thalamus | - | 2.2/2.4 | 7.5 (-3, -17, 8)/7.5 (6, -25, 7) |
|  |  |  |  |  |  |
| IC19 | Positive | Medial Frontal Gyrus | 6, 8, 9, 10 | 3.8/2.8 | 8.6 (-3, 58, 21)/8.6 (3, 58, 21) |
|  |  | Superior Frontal Gyrus | 9, 10 | 1.3/* | 7.2 (-4, 56, 25)/* |
|  |  |  |  |  |  |
| IC20 | Positive | Lentiform Nucleus | - | 1.3/1.5 | 4.3 (-18, 5, -2)/4.3 (19, 3, -7) |
|  |  |  |  |  |  |
| IC21 | Positive | Middle Temporal Gyrus | 19, 39 | 3.1/1.4 | 6.7 (-37, -74, 20)/5.4 (45, -64, 16) |
|  |  | Posterior Cingulate | 23, 29, 30, 31 | 2.0/1.2 | 6.5 (-1, -57, 12)/5.4 (4, -57, 12) |
|  |  |  |  |  |  |
| IC22 | Positive | Superior Frontal Gyrus | 9, 10 | 1.5/3.9 | 6.7 (-33, 51, 16)/7.9 (30, 57, 14) |
|  |  | Middle Frontal Gyrus | 9, 10, 46 | 3.6/3.5 | 7.1 (-31, 54, 12)/7.6 (28, 58, 10) |
|  |  |  |  |  |  |
| IC23 | Positive | Superior Temporal Gyrus | 13, 22, 39 | 4.0/2.8 | 5.8 (-53, -54, 16)/6.3 (56, -48, 13) |
|  |  | Middle Temporal Gyrus | 19, 21, 22, 39 | 2.6/2.0 | 5.7 (-53, -56, 12)/5.2 (56, -50, 6) |
|  |  |  |  |  |  |
| IC24 | Positive | Postcentral Gyrus | 40 | */1.3 | */6.2 (58, -28, 20) |
|  |  | Inferior Parietal Lobule | 40 | */1.7 | */5.8 (59, -30, 24) |
|  |  | Superior Temporal Gyrus | 22, 41, 42 | */1.1 | */5.6 (58, -28, 16) |
|  |  | Inferior Frontal Gyrus | 44, 46, 47 | */1.3 | */5.4 (45, 42, 7) |
|  |  |  |  |  |  |
| IC25 | Positive | Inferior Frontal Gyrus | 45, 47 | 1.5/1.3 | 6.8 (-43, 15, -4)/5.8 (46, 16, -3) |
|  |  | Insula | 13 | 2.0/* | 6.6 (-46, 12, -2)/* |
|  |  | Superior Frontal Gyrus | 6, 8 | 1.0/* | 5.4 (0, 9, 49)/* |
|  |  |  |  |  |  |
| IC26 | Positive | Medial Frontal Gyrus | 6, 8, 9 | 1.9/1.4 | 5.9 (-4, 46, 36)/6.0 (4, 47, 36) |
|  |  | Superior Frontal Gyrus | 8, 9 | 1.2/* | 5.0 (-1, 41, 44)/* |
|  |  |  |  |  |  |
| IC27 | Positive | Cingulate Gyrus | 24, 31, 32 | 1.7/1.3 | 5.5 (0, 12, 33)/4.7 (4, 14, 32) |
|  |  |  |  |  |  |
| Note: L/R: left/right; *: non-significant voxels or voxels with *z<*3.5 and volume smaller than 1cm^3^ | | | | | |

| Table S2.1.3. Independent components determined by minimum description length criteria for the N-back task. | | | | | |
| --- | --- | --- | --- | --- | --- |
| Component | Voxel directionality | Brain region labels | Brodmann area | L/R volumes (cm^3^) | L/R: max z-value (x,y,z) |
| IC1 | Positive | Lingual Gyrus | 17, 18, 19 | 4.4/4.3 | 8.2 (-1, -75, 0)/8.1 (3, -72, 4) |
|  |  | Cuneus | 17, 18, 23, 30 | 2.7/3.3 | 7.6 (-1, -72, 7)/7.8 (4, -72, 7) |
|  |  |  |  |  |  |
| IC2 | Positive | Precuneus | 7, 23, 31 | 3.6/3.4 | 8.5 (0, -68, 27)/7.6 (4, -68, 27) |
|  |  | Cuneus | 7, 18, 19 | 1.2/* | 7.0 (-3, -72, 33)/* |
|  |  | Posterior Cingulate | 23, 29, 30, 31 | 1.7/1.5 | 6.8 (0, -44, 22)/5.9 (4, -59, 25) |
|  |  | Cingulate Gyrus | 23, 31 | 1.3/* | 6.3 (0, -59, 27)/* |
|  |  |  |  |  |  |
| IC3 | Positive | Anterior Cingulate | 10, 24, 32 | 2.4/2.8 | 8.2 (-4, 44, -2)/8.9 (4, 43, 0) |
|  |  | Medial Frontal Gyrus | 10 | 1.5/1.4 | 7.7 (-1, 49, 1)/7.7 (6, 51, -4) |
|  |  |  |  |  |  |
| IC4 | Positive | Superior Temporal Gyrus | 13, 21, 22, 41, 42 | 4.7/4.3 | 6.7 (-55, -20, 7)/7.1 (56, -11, -6) |
|  |  | Precentral Gyrus | 3, 6, 13, 42, 43 | 1.0/1.7 | 5.2 (-56, -11, 10)/6.4 (49, -12, 6) |
|  |  | Transverse Temporal Gyrus | 41, 42 | 1.0/* | 5.7 (-56, -19, 10)/* |
|  |  | Insula | 13 | 1.5/1.9 | 5.6 (-46, -12, 4)/5.7 (46, -15, 6) |
|  |  |  |  |  |  |
| IC5 | Positive | Superior Temporal Gyrus | 21, 22, 38 | 2.6/2.1 | 12.6 (-40, 4, -15)/12.1 (40, 7, -15) |
|  |  | Sub-Gyral | 13, 21 | 2.1/1.4 | 10.7 (-43, -1, -10)/9.9 (43, 1, -10) |
|  |  | Inferior Frontal Gyrus | 13, 47 | 1.1/1.3 | 9.5 (-39, 13, -14)/9.2 (37, 14, -16) |
|  |  |  |  |  |  |
| IC6 | Positive | Extra-Nuclear | - | 1.8/1.4 | 7.3 (-4, -27, 7)/8.2 (4, -25, 7) |
|  |  | Thalamus | - | 2.0/2.4 | 6.9 (-4, -22, 7)/7.6 (4, -21, 8) |
|  |  | Lateral Ventricle | - | 1.0/* | 6.7 (-3, 1, 7)/* |
|  |  |  |  |  |  |
| IC7 | Positive | Medial Frontal Gyrus | 8, 9, 10 | 3.7/3.6 | 8.0 (-3, 61, 17)/7.5 (3, 60, 21) |
|  |  | Superior Frontal Gyrus | 9, 10 | 1.5/1.3 | 6.4 (-4, 56, 25)/6.0 (4, 57, 25) |
|  |  | Anterior Cingulate | 24, 32 | 1.0/* | 5.9 (0, 35, 20)/* |
|  |  |  |  |  |  |
| IC8 | Positive | Culmen | - | 2.8/2.6 | 15.3 (-3, -39, -6)/14.7 (1, -39, -6) |
|  |  |  |  |  |  |
| IC9 | Negative | Inferior Frontal Gyrus | 9, 44, 45, 46, 47 | */3.1 | */7.0 (53, 28, 6) |
|  |  | Precentral Gyrus  Middle Frontal Gyrus  Superior Temporal Gyrus  Middle Temporal Gyrus | 6, 43, 44  9, 10, 46  21, 22, 39, 42  19, 21, 22, 39 | */1.3  */1.0  */2.8  */1.3 | */6.5 (58, 11, 12)  */5.4 (48, 38, -5)  */5.1 (61, -8, 6)  */4.9 (64, -36, 4) |
|  | Positive | Inferior Frontal Gyrus | 9, 44, 45, 46, 47 | 3.1/* | 8.8 (-48, 37, 7)/* |
|  |  | Medial Frontal Gyrus | 6, 8, 9, 10, 46 | 3.2/* | 8.1 (-48, 33, 15)/* |
|  |  | Postcentral Gyrus  Precentral Gyrus  Superior Temporal Gyrus | 2, 3, 40, 43  6, 9, 43, 44  21, 22, 38, 42 | 1.0/*  2.0/*  3.1/* | 7.5 (-62, -23, 16)/*  7.3 (-58, 2, 7)/*  7.2 (-62, -28, 16)/* |
|  |  |  |  |  |  |
| IC10 | Positive | Postcentral Gyrus | 1, 2, 3, 40 | 3.6/3.6 | 8.0 (-40, -27, 53)/7.0 (40, -23, 53) |
|  |  | Precentral Gyrus  Inferior Parietal Lobule  Sub-Gyral | 4, 6  2, 40  40 | 1.4/1.9  3.6/1.3  1.0/* | 7.3 (-37, -24, 55)/6.6 (37, -26, 55)  7.1 (-42, -35, 51)/5.4 (48, -28, 47)  6.5 (-37, -31, 46)/* |
|  |  |  |  |  |  |
| IC11 | Positive | Middle Occipital Gyrus | 18, 19, 37 | 2.6/1.8 | 9.7 (-25, -84, 8)/7.4 (30, -77, 17) |
|  |  | Cuneus | 7, 17, 18, 19 | 3.9/2.8 | 9.6 (-25, -82, 11)/7.1 (27, -77, 20) |
|  |  | Precuneus | 7, 19, 31 | 1.3/1.0 | 7.7 (-24, -78, 26)/6.2 (22, -77, 26) |
|  |  | Lingual Gyrus | 17, 18 | 1.5/* | 7.7 (-21, -85, 3)/* |
|  |  | Sub-Gyral | - | 1.3/1.4 | 7.6 (-25, -84, 1)/6.7 (28, -76, 24) |
|  |  |  |  |  |  |
| IC12 | Positive | Inferior Parietal Lobule | 7, 39, 40 | */2.5 | */6.5 (43, -57, 42) |
|  |  | Middle Frontal Gyrus | 6, 8, 9, 10, 46 | */6.9 | */6.4 (30, 59, 8) |
|  |  | Superior Frontal Gyrus | 6, 8, 10 | */3.1 | */5.7 (25, 61, 10) |
|  |  | Supramarginal Gyrus | 40 | */1.9 | */5.2 (50, -52, 36) |
|  |  |  |  |  |  |
| IC13 | Positive | Declive | - | 6.4/4.8 | 7.7 (-24, -72, -20)/5.9 (25, -70, -20) |
|  |  | Uvula | - | 1.5/* | 7.0 (-25, -71, -23)/* |
|  |  | Pyramis | - | 1.3/1.0 | 6.2 (-24, -69, -27)/5.2 (21, -68, -27) |
|  |  |  |  |  |  |
| IC14 | Positive | Precuneus | 7, 19, 39 | 1.5/* | 7.0 (-33, -66, 36)/* |
|  |  | Inferior Parietal Lobule | 39, 40 | 1.9/* | 6.7 (-36, -63, 38)/* |
|  |  | Sub-Gyral | * | 2.8/* | 6.5 (-33, -66, 33)/* |
|  |  | Angular Gyrus  Inferior Frontal Gyrus  Middle Frontal Gyrus  Supramarginal Gyrus | 39  9, 46  9, 10, 46  40 | 1.2/*  2.2/*  1.9/*  1.4/* | 6.1 (-33, -60, 34)/*  5.8 (-46, 16, 25)/*  5.8 (-45, 21, 23)/*  5.0 (-43, -53, 36)/* |
|  |  |  |  |  |  |
| IC15 | Positive | Postcentral Gyrus | 3, 5, 40 | */1.0 | */6.2 (10, -40, 63) |
|  |  | Paracentral Lobule | 4, 5, 6, 7 | 1.1/1.2 | 8.3 (-3, -43, 61)/7.5 (4, -31, 64) |
|  |  | Precuneus | 7 | 1.3/1.0 | 8.1 (-1, -49, 58)/6.7 (3, -49, 56) |
|  |  | Medial Frontal Gyrus  Superior Frontal Gyrus  Precentral Gyrus  Middle Frontal Gyrus | 6  6, 8  4, 6  6, 8 | 1.1/1.1  2.9/3.1  1.5/1.4  1.2/1.5 | 7.4 (-3, -27, 64)/6.9 (4, -25, 64)  6.5 (-1, 19, 56)/6.3 (4, 6, 60)  5.0 (-19, -28, 64)/5.5 (12, -31, 64)  4.6 (-16, 9, 58)/4.7 (30, 11, 55) |
|  |  |  |  |  |  |
| IC16 | Positive | Declive | - | 2.0/1.0 | 8.2 (-33, -75, -21)/6.0 (34, -71, -22) |
|  |  |  |  |  |  |
| IC17 | Positive | Posterior Cingulate | 23, 29, 30, 31 | 2.9/2.4 | 9.0 (-3, -57, 8)/8.2 (4, -54, 10) |
|  |  |  |  |  |  |
| IC18 | Positive | Culmen | - | 2.8/2.0 | 13.0 (-12, -37, -17)/11.5 (12, -37, -17) |
|  |  |  |  |  |  |
| IC19 | Positive | Superior Temporal Gyrus | 22, 38, 39 | 2.8/1.3 | 7.6 (-52, -58, 16)/4.3 (55, -50, 14) |
|  |  | Middle Temporal Gyrus  Inferior Frontal Gyrus  Supramarginal Gyrus | 19, 21, 37, 39  44, 45, 47  40 | 5.9/*  1.6/*  1.2/* | 7.5 (-52, -59, 12)/*  6.1 (-49, 16, -4)/*  5.9 (-55, -51, 23)/* |
|  |  |  |  |  |  |
| IC20 | Positive | Insula | 13, 22 | 2.2/1.5 | 6.3 (-42, -13, -1)/5.7 (43, -5, -3) |
|  |  |  |  |  |  |
| IC21 | Positive | Precuneus | 7 | 4.4/4.2 | 8.5 (-1, -66, 44)/8.3 (3, -67, 42) |
|  |  |  |  |  |  |
| IC22 | Positive | Superior Frontal Gyrus | 8, 9 | 2.8/1.2 | 7.0 (-1, 53, 32)/5.8 (3, 54, 30) |
|  |  | Medial Frontal Gyrus | 6, 8, 9 | 2.9/1.9 | 6.7 (-1, 49, 34)/6.1 (3, 53, 34) |
|  |  | Middle Frontal Gyrus | 8, 9 | 3.4/* | 4.8 (-43, 16, 40)/* |
|  |  |  |  |  |  |
| IC23 |  | None |  |  |  |
|  |  |  |  |  |  |
| IC24 | Positive | Inferior Frontal Gyrus | 45, 47 | 1.3/2.7 | 5.9 (-43, 13, -4)/6.4 (43, 18, -4) |
|  |  | Insula | 13 | 1.2/1.0 | 5.5 (-43, 14, -1)/5.5 (40, 15, -2) |
|  |  | Cingulate Gyrus | 24, 32 | 1.2/1.0 | 4.9 (-1, 11, 40)/5.2 (1, 14, 40) |
|  |  |  |  |  |  |
| IC25 | Positive | Paracentral Lobule | 5, 6, 31 | 1.0/1.0 | 4.5 (0, -25, 49)/4.6 (1, -39, 50) |
|  |  | Precuneus | 7 | 1.6/* | 4.4 (0, -44, 50)/* |
|  |  |  |  |  |  |
| IC26 |  | None |  |  |  |
|  |  |  |  |  |  |
| Note: L/R: left/right; *: non-significant voxels or voxels with *z<*3.5 and volume smaller than 1cm^3^ | | | | | |

| Table S2.1.4. Independent components determined by minimum description length criteria for the Oddball task. | | | | | |
| --- | --- | --- | --- | --- | --- |
| Component | Voxel directionality | Brain region labels | Brodmann area | L/R volumes (cm^3^) | L/R: max z-value (x,y,z) |
| IC1 | Positive | Culmen | - | 2.9/2.9 | 13.8 (-6, -38, -8)/14.0 (6, -39, -8) |
|  |  |  |  |  |  |
| IC2 | Positive | Superior Temporal Gyrus | 13, 21, 22, 38 | 2.2/1.9 | 13.8 (-39, 6, -15)/13.8 (40, 7, -15) |
|  |  | Sub-Gyral | 13, 21 | 2.0/1.3 | 11.7 (-42, -2, -12)/10.6 (43, 1, -10) |
|  |  | Inferior Frontal Gyrus | 13, 47 | */1.4 | */10.5 (37, 14, -16) |
|  |  |  |  |  |  |
| IC3 | Positive | Lingual Gyrus | 18, 19 | 3.1/3.8 | 11.1 (-3, -78, 2)/11.9 (3, -78, 2) |
|  |  | Cuneus | 17, 18, 23, 30 | 2.5/2.9 | 10.5 (-1, -75, 6)/11.4 (3, -76, 6) |
|  |  |  |  |  |  |
| IC4 | Positive | Anterior Cingulate | 10, 24, 32 | 2.2/2.8 | 7.3 (-3, 43, 3)/8.1 (3, 43, 3) |
|  |  | Medial Frontal Gyrus | 10 | 2.2/2.0 | 7.1 (-3, 53, -4)/7.5 (3, 53, -4) |
|  |  | Superior Frontal Gyrus | 10 | */1.6 | */5.5 (6, 56, -1) |
|  |  |  |  |  |  |
| IC5 | Positive | Lentiform Nucleus | - | 1.1/1.7 | 4.3 (-19, 6, -4)/4.4 (18, 6, -9) |
|  |  |  |  |  |  |
| IC6 | Positive | Superior Temporal Gyrus | 13, 22, 41, 42 | 4.7/3.1 | 6.0 (-55, -21, 8)/6.5 (56, -9, 6) |
|  |  | Insula | 13, 22 | 1.2/1.6 | 5.2 (-46, -11, 4)/5.5 (46, -12, 6) |
|  |  | Precentral Gyrus | 6, 13, 43 | */1.0 | */5.7 (49, -9, 6) |
|  |  |  |  |  |  |
| IC7 | Positive | Postcentral Gyrus | 1, 2, 3, 40 | 3.9/3.4 | 7.5 (-40, -28, 51)/6.6 (42, -23, 51) |
|  |  | Inferior Parietal Lobule | 40 | 4.1/1.8 | 7.1 (-45, -35, 46)/5.5 (42, -28, 42) |
|  |  | Sub-Gyral | - | 1.2/0.7 | 6.8 (-40, -34, 46)/5.2 (39, -31, 44) |
|  |  | Precentral Gyrus | 4, 6 | 1.2/1.7 | 6.2 (-36, -23, 55)/5.8 (39, -20, 53) |
|  |  |  |  |  |  |
| IC8 | Positive | Medial Frontal Gyrus | 9, 10 | 3.3/3.1 | 7.7 (-4, 55, 17)/7.4 (4, 57, 17) |
|  |  | Anterior Cingulate | 10, 24, 32 | 1.2/* | 6.5 (0, 36, 20)/* |
|  |  |  |  |  |  |
| IC9 | Positive | Inferior Frontal Gyrus | 45, 47 | 1.3/2.1 | 5.7 (-42, 16, -3)/6.1 (45, 18, -3) |
|  |  | Insula | 13 | 1.3/1.0 | 5.4 (-40, 17, 1)/5.0 (37, 18, 1) |
|  |  |  |  |  |  |
| IC10 | Positive | Precuneus | 7, 23, 31, 39 | 3.8/2.0 | 9.1 (-1, -67, 27)/8.1 (1, -70, 27) |
|  |  | Posterior Cingulate | 23, 29, 30, 31 | 2.6/1.9 | 7.4 (0, -52, 21)/6.6 (3, -60, 23) |
|  |  | Cingulate Gyrus | 23, 31 | 1.2/* | 7.2 (-1, -58, 27)/* |
|  |  |  |  |  |  |
| IC11 | Positive | Middle Temporal Gyrus | 19, 21, 37, 39 | 3.8/3.3 | 6.3 (-52, -56, 10)/5.9 (55, -49, 8) |
|  |  | Superior Temporal Gyrus | 22, 39 | 2.6/2.9 | 5.8 (-52, -57, 14)/6.1 (55, -48, 12) |
|  |  |  |  |  |  |
| IC12 | Positive | Cuneus | 7, 17, 18, 19, 23 | 4.0/3.7 | 11.9 (0, -75, 28)/11.5 (4, -75, 30) |
|  |  | Precuneus | 7, 31 | 2.1/2.7 | 10.0 (-3, -72, 28)/9.3 (1, -71, 28) |
|  |  |  |  |  |  |
| IC13 | Positive | Extra-Nuclear | - | 1.0/1.2 | 8.8 (-3, -27, 7)/7.6 (4, -28, 9) |
|  |  | Thalamus | - | 1.7/1.7 | 6.6 (-4, -19, 8)/8.3 (4, -22, 7) |
|  |  |  |  |  |  |
| IC14 |  | None |  |  |  |
|  |  |  |  |  |  |
| IC15 | Positive | Precuneus | 7, 19, 39 | 1.5/* | 6.8 (-34, -66, 38)/* |
|  |  | Inferior Parietal Lobule | 39, 40 | 1.9/* | 6.5 (-37, -63, 38)/* |
|  |  | Middle Frontal Gyrus | 9, 10, 46 | 3.1/* | 6.1 (-46, 25, 23)/* |
|  |  | Sub-Gyral | - | 2.8/* | 5.9 (-31, -68, 31)/* |
|  |  | Angular Gyrus | 39 | 1.0/* | 5.9 (-37, -60, 34)/* |
|  |  | Inferior Frontal Gyrus | 9, 45, 46 | 2.9/* | 5.6 (-43, 36, 13)/* |
|  |  | Supramarginal Gyrus | 40 | 1.3/* | 5.0 (-40, -50, 36)/* |
|  |  |  |  |  |  |
| IC16 | Positive | Superior Frontal Gyrus | 9, 10 | 1.4/4.3 | 6.2 (-30, 52, 16)/7.7 (30, 57, 14) |
|  |  | Middle Frontal Gyrus | 8, 9, 10 | 2.8/3.8 | 6.6 (-30, 54, 12)/7.4 (28, 58, 10) |
|  |  |  |  |  |  |
| IC17 | Positive | Lingual Gyrus | 18, 19 | 1.7/1.7 | 9.0 (-10, -58, 3)/8.3 (12, -55, 5) |
|  |  | Culmen | - | 1.5/1.2 | 8.5 (-9, -53, 1)/7.8 (10, -52, 1) |
|  |  | Posterior Cingulate | 23, 29, 30, 31 | 2.6/2.9 | 7.5 (-12, -60, 10)/7.9 (13, -56, 8) |
|  |  |  |  |  |  |
| IC18 | Positive | Middle Temporal Gyrus | 19, 39 | 2.4/* | 7.2 (-50, -63, 22)/* |
|  |  | Medial Frontal Gyrus | 6, 8, 9, 10 | 2.0/* | 6.9 (-3, 50, 34)/* |
|  |  | Superior Frontal Gyrus | 6, 8, 9 | 5.1/* | 6.8 (-3, 53, 31)/* |
|  |  | Superior Temporal Gyrus | 22, 39 | 1.5/* | 6.7 (-53, -60, 21)/* |
|  |  | Middle Frontal Gyrus | 6, 8, 9 | 2.3/* | 5.3 (-42, 16, 42)/* |
|  |  |  |  |  |  |
| IC19 | Positive | Middle Occipital Gyrus | 19 | 3.3/2.7 | 8.2 (-27, -81, 11)/7.6 (31, -78, 11) |
|  |  | Cuneus | 7, 17, 18, 19 | 2.2/1.2 | 7.4 (-22, -82, 11)/6.6 (27, -79, 13) |
|  |  | Sub-Gyral | - | 1.0/1.6 | 6.4 (-25, -77, 22)/6.5 (30, -74, 22) |
|  |  | Declive | - | 1.2/1.7 | 4.8 (-28, -73, -17)/4.9 (30, -70, -17) |
|  |  |  |  |  |  |
| IC20 | Positive | Insula | 13, 22 | 2.0/1.4 | 6.6 (-43, -7, -3)/6.2 (45, -5, -3) |
|  |  | Superior Temporal Gyrus | 13, 22, 38 | 1.0/1.1 | 5.2 (-48, -5, -3)/5.9 (45, -1, -5) |
|  |  |  |  |  |  |
| IC21 | Positive | Inferior Parietal Lobule | 40 | 1.7/3.6 | 5.1 (-58, -33, 29)/6.2 (61, -27, 27) |
|  |  | Postcentral Gyrus | 2, 40 | */1.8 | */5.7 (61, -23, 21) |
|  |  |  |  |  |  |
| IC22 | Positive | Paracentral Lobule | 4, 5, 6, 7 | 2.3/1.9 | 8.1 (-1, -46, 59)/7.4 (3, -30, 64) |
|  |  | Medial Frontal Gyrus | 6 | 1.3/1.4 | 7.2 (-3, -25, 64)/7.2 (3, -25, 64) |
|  |  | Precuneus | 7 | 1.1/* | 6.9 (-4, -49, 58)/* |
|  |  | Postcentral Gyrus | 3, 4, 5, 40 | 1.2/1.0 | 4.8 (-10, -36, 63)/4.9 (10, -40, 63) |
|  |  |  |  |  |  |
| IC23 | Positive | Inferior Parietal Lobule | 7, 39, 40 | 0.6/2.6 | 4.1 (-45, -59, 40)/8.9 (45, -59, 40) |
|  |  | Angular Gyrus | 39 | */1.0 | 3.9 (-45, -62, 36)/8.6 (45, -62, 36) |
|  |  | Supramarginal Gyrus | 40 | */2.6 | */7.4 (50, -49, 37) |
|  |  | Middle Temporal Gyrus | 39 | */1.1 | */6.7 (46, -62, 29) |
|  |  | Middle Frontal Gyrus | 6, 8, 9, 10, 46 | */4.2 | */5.3 (42, 20, 41) |
|  |  | Superior Frontal Gyrus | 8, 10 | */1.2 | */5.0 (33, 19, 49) |
|  |  |  |  |  |  |
| IC24 | Positive | Precuneus | 7 | 5.1/4.5 | 11.7 (0, -61, 47)/12.1 (1, -58, 51) |
|  |  |  |  |  |  |
| IC25 | Negative | Culmen | - | 2.0/1.5 | 5.5 (-10, -54, -4)/4.7 (13, -49, -4) |
|  |  |  |  |  |  |
| IC26 | Positive | - | - | 1.7/1.4 | 9.5 (0, -46, -23)/8.0 (4, -46, -26) |
|  |  | Nodule | - | */1.0 | */9.2 (0, -49, -30) |
|  |  | Culmen | - | 2.2/2.0 | 6.8 (-3, -49, -19)/7.1 (3, -49, -19) |
|  |  | Declive | - | 4.1/4.5 | 5.6 (-4, -66, -19)/5.7 (4, -66, -19) |
|  |  | Uvula | - | 1.0/* | 5.2 (-22, -69, -23)/* |
|  |  |  |  |  |  |
| IC27 | Positive | Middle Frontal Gyrus | 9, 46 | */2.2 | */6.6 (48, 28, 19) |
|  |  | Inferior Frontal Gyrus | 9, 44, 45, 46 | 1.2/3.6 | 4.7 (-45, 13, 23)/6.4 (48, 23, 21) |
|  |  | Sub-Gyral | - | 1.1/2.0 | 4.9 (-45, 18, 21)/6.0 (43, 26, 19) |
|  |  |  |  |  |  |
| Note: L/R: left/right; *: non-significant voxels or voxels with *z<*3.5 and volume smaller than 1cm^3^ | | | | | |

| Table S2.1.5. Independent components determined by minimum description length criteria for the Go-NoGo task. | | | | | |
| --- | --- | --- | --- | --- | --- |
| Component | Voxel directionality | Brain region labels | Brodmann area | L/R volumes (cm^3^) | L/R: max z-value (x,y,z) |
| IC1 |  | None |  |  |  |
|  |  |  |  |  |  |
| IC2 | Positive | Culmen | - | 2.4/2.0 | 11.5 (-9, -39, -11)/10.7 (9, -39, -10) |
|  |  |  |  |  |  |
| IC3 | Positive | Declive | - | 3.3/3.6 | 6.3 (-4, -69, -17)/6.5 (0, -67, -17) |
|  |  | Culmen | - | 1.2/1.2 | 4.8 (-19, -59, -21)/4.3 (12, -56, -17) |
|  |  |  |  |  |  |
| IC4 | Positive | Precuneus | 7, 19, 39 | 2.1/* | 9.1 (-34, -66, 40)/* |
|  |  | Inferior Parietal Lobule | 39, 40 | 1.7/* | 8.5 (-37, -63, 40)/* |
|  |  | Angular Gyrus | 39 | 1.3/* | 7.4 (-34, -68, 33)/* |
|  |  | Sub-Gyral | - | 1.7/* | 7.0 (-31, -71, 31)/* |
|  |  | Supramarginal Gyrus | - | 1.0/* | 4.9 (-43, -52, 36)/* |
|  |  |  |  |  |  |
| IC5 | Positive | Precuneus | 7, 23, 31 | 2.6/1.2 | 8.0 (0, -65, 27)/6.5 (4, -65, 27) |
|  |  | Posterior Cingulate | 23, 29, 30, 31 | 1.6/1.3 | 7.3 (0, -51, 21)/6.2 (3, -49, 25) |
|  |  | Cingulate Gyrus | 31 | 1.1/* | 6.9 (0, -61, 29)/* |
|  |  |  |  |  |  |
| IC6 | Positive | Superior Temporal Gyrus | 22, 39 | 1.7/* | 8.5 (-49, -61, 20)/* |
|  |  | Middle Temporal Gyrus | 19, 39 | 3.0/* | 8.3 (-50, -60, 23)/* |
|  |  | Supramarginal Gyrus | 40 | 1.3/* | 6.9 (-50, -58, 31)/* |
|  |  |  |  |  |  |
| IC7 | Positive | Precuneus | 7, 31 | 3.6/2.6 | 7.9 (-1, -64, 44)/7.2 (1, -62, 42) |
|  |  |  |  |  |  |
| IC8 |  | None |  |  |  |
|  |  |  |  |  |  |
| IC9 | Positive | Inferior Parietal Lobule | 7, 39, 40 | */2.7 | */7.7 (43, -59, 40) |
|  |  | Supramarginal Gyrus | 40 | */2.5 | */7.4 (49, -56, 32) |
|  |  | Superior Temporal Gyrus | 22, 39 | */1.6 | */7.0 (50, -56, 29) |
|  |  |  |  |  |  |
| IC10 | Positive | Superior Temporal Gyrus | 13, 22, 41, 42 | 4.2/1.7 | 7.2 (-53, -22, 8)/6.6 (55, -19, 8) |
|  |  |  |  |  |  |
| IC11 | Positive | Cuneus | 17, 18 | 1.4/* | 6.2 (-25, -81, 10)/* |
|  |  | Middle Occipital Gyrus | - | 1.2/* | 5.9 (-25, -82, 6)/* |
|  |  | Declive | - | 1.2/* | 5.5 (-31, -73, -17)/* |
|  |  |  |  |  |  |
| IC12 | Positive | Paracentral Lobule | 4, 5, 6, 7 | 1.4/1.2 | 5.5 (0, -39, 59)/5.0 (1, -39, 55) |
|  |  | Precuneus | 7 | 1.3/* | 5.4 (0, -49, 52)/* |
|  |  |  |  |  |  |
| IC13 | Positive | Cuneus | 7, 17, 18, 19, 23, 30 | 2.8/2.6 | 6.4 (-1, -81, 11)/6.9 (1, -77, 19) |
|  |  | Lingual Gyrus | 18 | 1.4/1.2 | 5.3 (-3, -75, 2)/6.1 (1, -77, 4) |
|  |  |  |  |  |  |
| IC14 | Positive | Posterior Cingulate | 23, 29, 30, 31 | 2.0/1.7 | 7.4 (-10, -57, 7)/6.5 (12, -55, 6) |
|  |  |  |  |  |  |
| IC15 | Positive | Inferior Parietal Lobule | 40 | 2.4/1.2 | 6.4 (-58, -35, 26)/5.4 (58, -32, 26) |
|  |  |  |  |  |  |
| IC16 | Positive | Inferior Parietal Lobule | 2, 40 | 3.4/* | 5.8 (-49, -32, 42)/* |
|  |  | Postcentral Gyrus | 1, 2, 3, 40 | 1.9/1.5 | 4.8 (-49, -27, 40)/4.1 (50, -27, 42) |
|  |  |  |  |  |  |
| IC17 |  | None |  |  |  |
|  |  |  |  |  |  |
| IC18 | Positive | Lingual Gyrus | 18, 19 | 1.6/1.1 | 4.9 (4, -74, -3)/4.7 (7, -71, -3) |
|  |  | Culmen | - | 1.1/* | 4.4 (-6, -64, -4)/* |
|  |  |  |  |  |  |
| IC19 |  | None |  |  |  |
|  |  |  |  |  |  |
| IC20 |  | None |  |  |  |
|  |  |  |  |  |  |
| IC21 |  | None |  |  |  |
|  |  |  |  |  |  |
| IC22 |  | None |  |  |  |
|  |  |  |  |  |  |
| Note: L/R: left/right; *: non-significant voxels or voxels with *z>*3.5 and volume smaller than 1cm^3^ | | | | | |

Table S2.2

*Intra-class correlations between MZ and DZ twins for the task-related independent components/regions-of-interest that show significant heritability.*

| **Task** | **IC / ROI** | **Contrast** | **ICC** | | **Starting**  **model** | **Final**  **model** |
| --- | --- | --- | --- | --- | --- | --- |
|  |  |  | **MZ** | **DZ** |  |  |

| Noncon  FEET | IC4 | Disgust >  Neutral | 0.222 | 0.278 | ACE | AE |
| --- | --- | --- | --- | --- | --- | --- |
|  | IC4 | Fear >  Happy | -0.076 | -0.027 | ACE | AE |
| N-back | IC4 | Target >  Baseline | 0.482 | 0.357 | ACE | AE |
|  | IC10 | Target > Baseline | 0.197 | 0.461 | ACE | AE |
|  | IC12 | NonTarget > Baseline | 0.193 | 0.279 | ACE | AE |
| Oddball | IC11 | Target > NonTarget | 0.340 | 0.193 | ACE | AE |
|  | IC23 | Target > NonTarget | 0.449 | -0.137 | ACE | AE |
| Noncon  FEET | L amygdala | Sad >  Neutral | 0.343 | 0.136 | ADE | AE |
|  | R amygdala | Sad > Neutral | 0.408 | -0.055 | ADE | AE |
| Oddball | Medial superior PFC | Target > NonTarget | 0.334 | 0.078 | ADE | AE |

*Note.* Noncon FEET = Nonconscious Facial Expressions of Emotion Task; IC = independent component; ROI = region-of-interest; L = left; R = right; PFC = prefrontal cortex; ICC = intra-class correlation; MZ = monozygotic; DZ = dizygotic.

Table S2.3

*Number of twin pairs included in the fMRI and twin modelling analyses for each task divided by sex and zygosity.*

| Task | Male | | | | Female | | | |
| --- | --- | --- | --- | --- | --- | --- | --- | --- |
|  | MZ pairs | | DZ pairs | | MZ pairs | | DZ pairs | |
| Nonconscious  FEET | 32 | 11 | | 45 | | 25 | |  |
| Conscious  FEET | 31 | 10 | | 44 | | 25 | |  |
| N-back | 23 | 10 | | 28 | | 23 | |  |
| Oddball | 31 | 10 | | 44 | | 24 | |  |
| Go-NoGo | 18 | 7 | | 25 | | 18 | |  |

*Note.* FEET = Nonconscious Facial Expressions of Emotion Task; MZ = monozygotic; DZ = dizygotic.
